# Reasoning in machine vision by learning fast and slow thinking

**DOI:** 10.1038/s41467-026-74579-8

**Published:** 2026-06-23

**Authors:** Shaheer U. Saeed, Yipei Wang, Veeru Kasivisvanathan, Brian R. Davidson, Matthew J. Clarkson, Yipeng Hu, Daniel C. Alexander

**Affiliations:** 1 Centre for Bioengineering, Queen Mary University of London, London, UK; 2 Digital Environment Research Institute, Queen Mary University of London, London, UK; 3 School of Engineering and Materials Science, Queen Mary University of London, London, UK; 4 UCL Hawkes Institute, University College London, London, UK; 5 Department of Medical Physics and Biomedical Engineering, University College London, London, UK; 6 Centre for Urology Imaging, Prostate, AI and Surgical Studies (COMPASS) Research Group, Division of Surgery and Interventional Science, University College London, London, UK; 7 Department of Urology, Comprehensive Cancer Center, Medical University of Vienna, Vienna, Austria; 8 Division of Surgery and Interventional Science, University College London, London, UK; 9 Department of Computer Science, University College London, London, UK

**Keywords:** Computer science, Biomedical engineering, Cancer imaging

## Abstract

Reasoning is a hallmark of human intelligence, enabling adaptive decision-making in complex unfamiliar scenarios. In contrast, machine intelligence remains bound to training data, unable to dynamically refine solutions at inference. While recent advances have explored machine reasoning - trading inference-time compute for improved performance - they focus on verbal domains such as mathematical problem-solving where explicit rules govern step-by-step solution generation. Many tasks lack sufficient labelled data and require alternative performance improvement mechanisms, such as inference-time compute. Here we present a paradigm for machine reasoning in vision, enabling performance improvements with increasing thinking time (inference-time compute), even with limited labelled data. Our approach is inspired by dual-process theories of human cognition, integrating a fast-thinking System I module for generating and verifying solutions in familiar tasks, with a slow-thinking System II module that iteratively refines predictions using self-play reinforcement learning, even when task-specific data is limited. This paradigm involves proposing, competing over, and refining solutions until convergence. We demonstrate that extended inference-time compute yields superior performance compared to large-scale supervised learning, foundation models, and human experts in vision tasks. These include computer-vision benchmarks and cancer localisation across five organs, highlighting the potential of inference-time compute for data-scarce problems.

## Abstract (web summary)

This work introduces a new approach to AI in which machines improve by “thinking” longer rather than learning from more data. This enables adaptable systems showing strong performance in challenging vision tasks like cancer detection.

## Introduction

Rethinking machine cognition - beyond data-driven learning: Human cognition excels at new tasks with minimal prior experience through reasoning, by dedicating cognitive resources, such as extended thinking time, to planning and strategising^1–3^. This is enabled by dual-process human cognition^1^: System I enables fast, intuitive decisions based on prior experience, while System II facilitates slow, deliberate reasoning, especially in unfamiliar scenarios. In the context of machines, reasoning or System II thinking is analogous to the ability to consume computational resources, such as inference-time compute, to improve performance, as opposed to using data for improvement, as noted in recent literature^4–6^. Conventional deep learning systems lack this capacity to perform reasoning or System II thinking^4,7^, relying heavily on extensive human-labelled data or repeated task exposure to perform well in familiar scenarios^8^. While such data-driven experience yields strong performance within the scope of training data, these systems falter when labelled data is insufficient to represent new tasks^8–10^. Here, we propose an algorithm which enables System II thinking and equips machines with the ability to improve performance with increasing thinking time (inference-time computation), rather than resorting to additional labelled data.

Two systems of human cognition and their transition in psychology: Theories from cognitive psychology have observed that fast and intuitive System I thinking allows humans to make confident decisions for familiar tasks, despite unclear immediate justifications^1–3^. In contrast, System II thinking consumes cognitive resources at the time of the decision-making^1^. For instance, a beginner chess player can reason about potential moves, plan and develop complex strategies using only the game rules, despite limited gameplay experience. One key cognitive resource consumed in such processes is thinking time, and its mechanisms include comparison of hypothetical scenarios, considering long-term outcomes in greater detail, and breaking problems into simpler parts before solving them^1,7^. Attention is another critical cognitive resource^1^, which can be consumed through mechanisms such as filtering out irrelevant information (e.g., ignoring background speech while reading)^11^. Consuming these resources allows improvements in decision quality, which is an ability considered unique to humans^7^.

With experience, humans can transition tasks from slow, deliberate System II to much faster System I thinking^3^ e.g., the ability of experienced chess players to execute strategies more quickly and instinctively as their experience grows. The interplay between fast and slow thinking allows versatility in human cognition, which is capable of handling both familiar and unfamiliar scenarios.

**Fig. 1:**
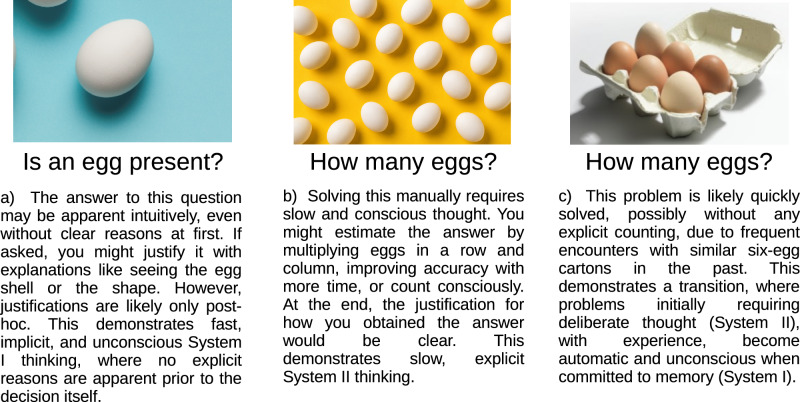
Examples illustrating System I and System II cognitive processes. **a**, **b** represent problems solved by System I and System II thinking, respectively. **c** represents a problem that has transitioned from System II to System I over time. Note: (**a**–**c**) are open stock photos available via an institutional Microsoft 365 subscription.

We present examples, in Fig. 1, showcasing tasks typically solved by System I and System II thinking, along with tasks that have likely transitioned between the two.

Shortfalls of machine cognition: Using the analogy of dual-process cognition, machines are limited in the sense that their predictions are largely feed-forward and made with fixed inference times, i.e., they operate only in a System I regime. In other words, supervised deep learning algorithms are highly dependent on large labelled data sets and lack mechanisms to improve by extending thinking time (inference-time compute)^4,7,10^, or handle scenarios beyond the training distribution^12^. Approaches like few-shot learning^13–15^ and foundation models^16–19^ aim to use task-specific data more efficiently. However, they exhibit fixed, often linear, computational complexity with respect to data availability, and the only viable mechanism to improve performance further is to train with more human-labelled data^20–22^.

The analogue for System II capabilities in machines would allow performance improvement with an increase in computation time, overcoming practical challenges stemming from the cost and scarcity of labelled data, as noted in recent literature^4–6,12,23–30^.

Progress towards inference-time compute capabilities in machines: There has been prior work implementing inference-time compute strategies for machine inference that are analogous to System II thinking. However, such approaches have largely focused on constrained and restrictive domains, such as board games^4^. These approaches modelled System I and System II separately, without explicit modelling of the connection between the two. In these works, the System II slow-thinking process is implemented as an exhaustive, structured, random or guided search over promising moves, as a mechanism to consume compute time^4,31,32^. While these approaches improve performance by extending compute time without requiring additional task-specific data, they are limited to scenarios with manageable search spaces or to specialised domains such as language^23,33–36^. Recent work has also modelled System II thinking using a reinforcement learning process guided by a reward signal. However, these remain limited to the language domain, relying largely on verifiable tasks such as mathematics problems, using rule-based verification of final outcomes or intermediate steps, to generate rewards^5,24,37,38^, sometimes involving human observers^39,40^.

Several existing approaches aim to refine initial visual predictions, marking a step toward inference-time compute. Structured-prediction methods such as CRF-based refinements^41^ enhance spatial coherence through hand-crafted constraints and a fixed number of update steps. Diffusion-based iterative approaches^42^ also refine predictions but follow predetermined generative trajectories, with fixed steps, and typically rely on large-scale datasets for training. Other methods improve data efficiency by selecting or adapting pretrained features^43^, or by refining internal representations through hierarchical modules^44,45^, though their inference-time computation is fixed once trained. Although System II reasoning operates during inference, it differs fundamentally from test-time adaptation approaches. Test-time adaptation typically updates the parameters of the predictive model to better match the test distribution. In contrast, in our framework, the System I networks remain fixed, while the System II module optimises a reinforcement learning policy that iteratively refines candidate segmentations. The optimisation therefore occurs in the solution space rather than through adaptation of the segmentation model parameters. Inference-time computation analogous to System II thinking would allow performance to improve with added unbounded computation/ thinking time, even when labelled data is scarce.

Another type of cognitive resource in machines is attention, which may also be considered as a model for System II thinking, although has been explored only in the context of natural language applications^46^. Techniques such as ‘divide-and-conquer’^47^ and ‘chain-of-thought’^6^ break down complex tasks into parts before solving them. These mechanisms enable neural networks to improve through a refinement of their inputs, either guided by a human observer^6^ or through iterative guided refinements of their own inputs, which also increases inference-time compute^46^. However, they remain limited to sequential query-response domains, such as large language models trained/fine-tuned on paired question-and-answer chat-based data, owing to the ease of verifying correctness in such domains. Arguably, attention mechanisms common in language applications do not model System II thinking^48–51^, as they do not possess the ability to dynamically alter behaviours for new samples or tasks, with attention often learnt based on task-specific experience itself.

Most of this previous work has focused on emulating verbal reasoning, however, non-verbal or visual reasoning has the potential to enhance various applications, including robotics, medical diagnostics, and astronomy. Many real-world tasks require reasoning about spatial relationships, object boundaries, and patterns without relying on language-based logic. In medical imaging, for instance, clinicians identify and interpret complex visual features to diagnose diseases–an inherently non-verbal reasoning process. Developing systems capable of such non-verbal reasoning is essential for advancing machine intelligence beyond text-based tasks and into broader, high-impact applications.

The main hindrance in implementing reasoning or inference-time computation for vision tasks, such as identifying object boundaries, stems from the fact that solutions are often more complex with much higher-dimensional solution spaces. The methods above do not extend naturally to explore such spaces, which often have nuanced solutions with multiple nearly-correct outcomes. This makes the development of the verification-based rewards challenging in such domains. For example, even in a simple real-world 2D image segmentation to localise objects, the solution space is astronomically large, far beyond the computational capacity for exhaustive search, or for manual design of rule-based verification strategies (as an example, assuming a 512 × 512 image with a single object, there are ${\sum }_{r=0}^{512\times 512}\frac{(512\times 512)!}{r!((512\times 512)-r)!}\gg 1{0}^{1000}$ possibilities; whereas for chess, 16 pieces per side, each with 16 moves, there are $\frac{16!}{1!(16-1)!}\times \frac{16!}{1!(16-1)!}=256$ possibilities per-move). Some efforts allow improved performance as a result of slow thinking^25,26^ but operate with fixed thinking periods, limiting their scalability, with further performance improvements not possible with an increase in thinking time. Thus, the modelling of the reasoning enabled by System II thinking remains uncharted in the context of vision applications.

**Fig. 2:**
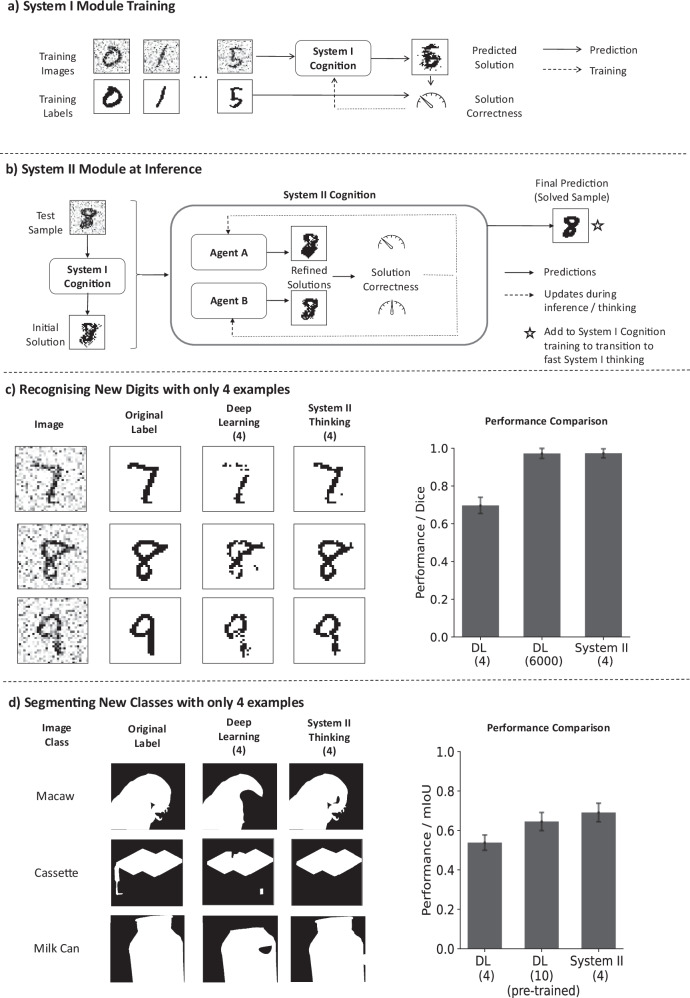
Overview of the proposed framework and its performance. **a**, **b** Present an overview of the training and evaluation of the proposed System II approach. **c**,** d** Show performance of the System II approach compared with common methods (numbers of samples used for training/ adaptation, from the target task, in brackets). Note: images in (**a**–**c**) are derived from the public domain MNIST dataset, the original ImageNet images are omitted from d) due to copyright; however, these and corresponding segmentations are available via the online repository: github.com/s-sd/system-II-vision^78^.

An auto-competing mechanism to enable inference-time compute: We propose two interconnected modules for enabling inference-time compute in machines, which are analogous to dual-process human cognition: (1) the System I module, trained on human-labelled data across various tasks, to produce fast solutions; and (2) the System II module, which allows for refinement and optimisation of solutions produced by the System I module. Even in data-limited scenarios, this framework allows performance improvements with added inference-time compute. Additionally, the System II module, after fully refining the solutions, feeds back to the System I module to improve its fast solutions for subsequent data, reducing the time required for refinement in the System II module. An overview of the described method is illustrated in Fig. 2a, b. A detailed description is presented in the ‘Methods’ section, and a mathematical treatment is presented in the supplementary materials ‘methodological description’.

## Results

### Demonstration in standard computer vision tasks

System II thinking recognises “new” digits with only four samples: Fig. 2a–c illustrates how the System II method offers substantial improvement over traditional supervised learning in a simple vision task. For the task of segmenting digits from noisy images, we trained the System I module with digits 0–5 (as shown in Fig. 2a), and then adapted this using only 4 samples from each of the unseen target digits 6–9. The System II module was then used to segment 4000 samples from each of the target digits 6–9 (as shown in Fig. 2b). Figure 2c shows that our dual-process approach, using extended thinking time to improve performance, substantially outperformed a deep learning algorithm (trained separately from the System I module but following a similar training scheme)^52^ trained and adapted with the same data. The System II approach, using only 4 samples from the target digits, also showed similar performance to a deep learning algorithm^52^ trained with 6000 samples from the target digits, representing an upper-bound performance in these new tasks.

System II thinking improves segmentation on new image classes: Fig. 2d illustrates the substantially improved segmentation performance of the System II method, on unseen real-world image classes. We trained the System I module to segment objects using 2000 images and labels randomly sampled from the ImageNet-S dataset^53^. We then adapted the System I module to segment 40 unseen target objects, using only 4 labelled samples for each. The System II module was then used to segment 6 unseen images for each of the 40 unseen objects. As shown in Fig. 2, System II thinking significantly outperformed the previous best deep-learning methods, not only when the deep learning methods used the same data, but also when they used substantially more pre-training data and labelled samples from the unseen target objects^54^.

Supplementary materials section `detailed results for computer vision tasks' shows that improvements offered by the System II approach were statistically significant, for both experiments above.

### Localisation of five types of abdomino-pelvic cancer

Motivation: Cancer detection and localisation in medical images, such as prostate magnetic resonance (MR) and liver computed tomography (CT), holds significant promise for non-invasive diagnosis, often even outperforming the invasive alternatives^55–57^. However, democratisation of these medical imaging technologies has been severely limited by the need for highly-specialised expertise and associated costs, often only accessible in a small number of large medical centres, even in the developed countries^10,55,58–61^. Deep learning has been effective in segmenting anatomical structures, such as organs, where variability is relatively constrained^17,52^. In contrast, cancer pathology presents categorically greater heterogeneity, with imaging manifestations that are less well-defined and continuously evolving due to new clinical discoveries and advancements in imaging technologies. This complexity is further compounded by the challenge of obtaining high-quality ground-truth labels, which require specialised clinical expertise or resource-intensive procedures such as independent histopathology examinations. Thus, cancer detection and localisation is a prime example where System I cognition falls short and System II cognition may offer data-efficient performance improvements that are comparable or even superior to what expert clinicians are capable of.

**Fig. 3:**
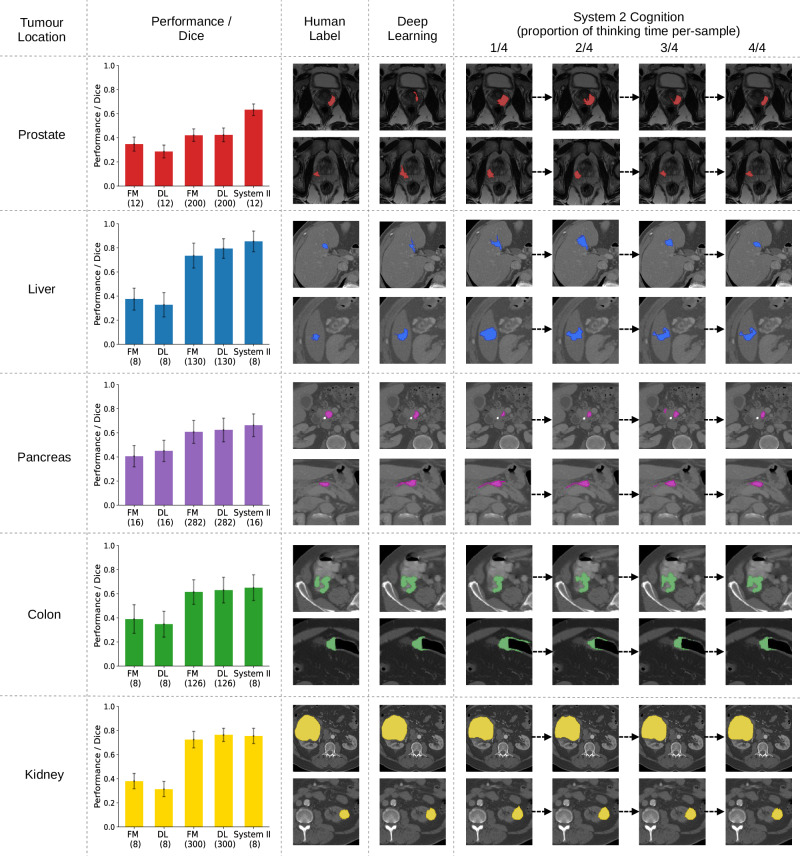
Performance of System II thinking compared with deep learning (DL) and foundation models (FM) in five cancer localisation tasks for different organs and image types. The left column shows statistically significant increases in cancer localisation performance compared to DL and FM with equivalent training data (indicated by the numbers in brackets below), and better or at least comparable performance even when DL and FM use substantially more training data. The remaining columns provide qualitative examples demonstrating the improvement as the inference-time computation increases, further discussed in the text.

System II sets a state-of-the-art performance for cancer localisation tasks: Fig. 3 shows that System II thinking achieved new state-of-the-art performance for cancer segmentation across all five distinct organs. The System I module was trained on abdomino-pelvic CT and MR images, from 955 3D human-labelled images, for over 13 anatomical structures, similar to prior work^62^ (summarised in Table 1 in supplementary materials). This is also markedly less than the pre-training data sets required in recent image foundation models^17^. This was then adapted using a few labelled samples (~ 8–16, specified where appropriate) to the unseen target tasks of cancer localisation on medical images. The System II thinking module was evaluated for each of the five clinical tasks of localising cancer on MR or CT images from real cancer patients. Compared to the previous best methods, the System II approach outperformed (percentage points reported here) cancer segmentation in the prostate^63–66^ by 20.7%, in the liver^67^ by 5.9%, in the pancreas^67^ by 3.9% and in the colon^67^ by 2.0%, with all improvements being statistically significant (all *p* − *values* < 0.001, paired Student’s *t* test at a significance level of *α *= 0.05). It is considered equivalent to the best-performing method for kidney cancer segmentation^68^. For this task, statistical significance was not found for the comparison (*p* − *value* = 0.051), with the intra-observer^69^ and inter-observer^70^ variabilities reported to be high, being similar to that between human and machine segmentation^69,70^, which limits the highest measurable performance for any manual or automated segmentation method, due to the limited detectable effect size.

System II thinking significantly outperforms foundation models: Fig. 3 also shows substantial improvements using System II thinking with only 8–16 labelled samples, compared to foundation models^71,72^ fine-tuned with the same or more (100–500) labelled samples. System II, using only 8–16 labelled samples, improves tumour segmentation over foundation models, using hundreds of labelled samples, by 20.9% in the prostate, 11.8% in the liver, 5.5% in the pancreas, 3.6% in the colon and 3.1% in the kidney, with all improvements being statistically significant (all *p* − *values* < 0.001).

Numerical results for all comparisons, for cancer localisation, are presented in Table 3 in the supplementary materials.

System II thinking outperforms radiologists with respect to independent histopathology ground-truth: Cancer presence in a subject can be identified through medical images and verified through an independent histopathology examination, for example, via a biopsy procedure. The classification of whether a patient has cancer or not, based on the medical image, has the potential to allow non-invasive diagnoses in lieu of any invasive histology-based confirmations. Thus, for the task of classifying cancer presence in patients using only medical images, the System II approach was compared with double-blind radiologists performing the same task. The patient-wise cancer presence classifications were verified, in terms of sensitivity and specificity, against histopathology examination results. The procedure for this evaluation is outlined in ‘Retrospective evaluation against human experts for prostate cancer detection’ in the supplementary materials. For this patient-wise classification, System II offered improved sensitivity (with 95% confidence interval) of 0.911 (0.880, 0.934) and specificity of 0.655 (0.576, 0.727) compared to the expert radiologist performance^55^ with a sensitivity of 0.88 (0.84–0.91) and a specificity of 0.45 (0.39–0.51), for the same task on the same data set.

### The abilities of the proposed inference-time compute framework

Our experiments focus on representative data-scarce vision tasks that are clinically and scientifically important, where conventional deep learning often struggles. We do not aim to cover the full breadth of visual reasoning. Instead, we show that inference-time computation, via competitive hypothesis refinement, can reliably improve performance when additional labelled data are limited or unavailable. These results illustrate a broader principle: increasing computation at inference can serve as an effective alternative to increasing training data, with potential to extend to other visual tasks in future work.

**Fig. 4:**
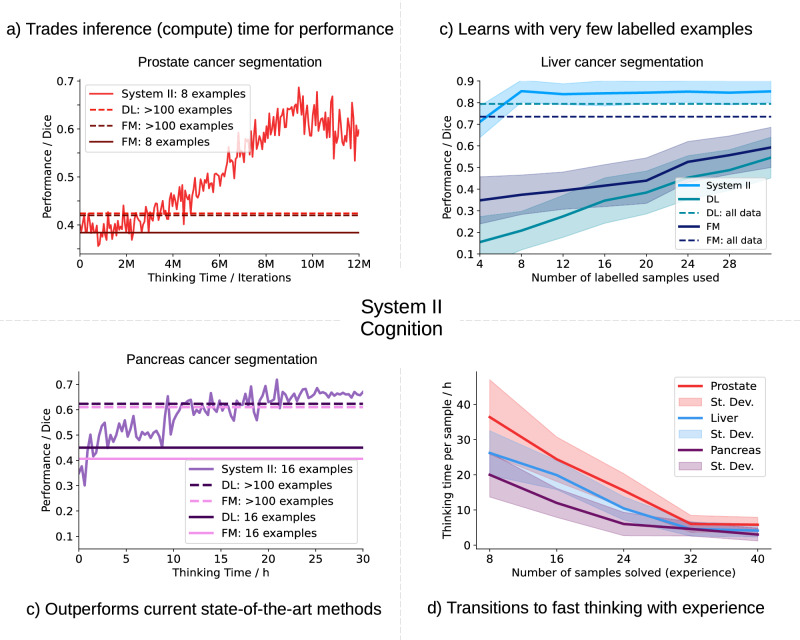
System II thinking. **a** improves performance with increasing thinking time, compared to supervised deep learning (DL) and foundation models (FM) with fixed inference time (measured in the unit of millions of optimisation iterations); (**b**) outperforms the current best-performing deep learning (DL) methods, using substantially fewer labelled task-specific samples; (**c**) attains peak performance with 8 labelled samples, compared to other methods requiring more than one hundred labelled samples; (**d**) transitions tasks to fast thinking, reducing thinking time as more unlabelled samples are fully refined (or solved) by System II thinking. Performance in plotted lines is the average across 20 samples from the test set. Complete results for all cancer types can be found in the `Comparison to existing methods for cancer localisation' section in the supplementary materials.

Trading inference-time compute for machine performance: Fig. 4a, b demonstrates a key feature of System II cognition - the ability to improve performance by utilising inference-time compute, to a much greater extent than alternative learning systems. This is shown as performance improves with a substantially longer period of increased thinking time, when we allow the System II module to auto-compete in an iterative manner, refining the solutions as the competition progresses. In this iterative refinement process, the space of performance improvement becomes much more expandable, compared with that from other single-pass or few-shot learning methods, such as foundation models that have a distinct aim of being data-efficient with fixed inference time.

Outperforming fixed-thinking-time System I approaches: Fig. 4a, b) show that the System II approach outperforms the currently best approaches in computer vision (full details and justifications are outlined in Table 3 in the supplementary materials), which all follow a fixed-thinking-time System I paradigm. The performance improvement can be seen despite the System II approach using less than ten times the amount of data required by the solitary System I data-driven approaches.

Learning with very few labelled samples: Fig. 4c shows that System II thinking achieves its peak performance using only 8 labelled samples. This trend, of achieving peak performance even with a small number of labelled samples, was observed for all tested applications, for all cancer localisation tasks, with only 8–16 samples (as shown in Table 3 in the supplementary materials). We report the System II performances at their peaks and compare these, not only to other methods (deep learning and foundation models) using equivalent amounts of data, but also to their converged peak performances, which require hundreds of samples.

Transitioning from slow System II to fast System I cognition: Fig. 4d shows the ability for the System II module to transition tasks to fast System I cognition. The System II module requires thinking time to refine solutions until no improvements are possible in the distribution-discriminator score, at which point a solution is considered fully refined and thus solved. This solved solution is used to re-train the System I module, which enables better initial solutions and more accurate distribution-discriminator scores for new samples and reduces subsequent thinking time in the System II stage. The ability of reducing thinking time while maintaining the same performance, without any new human-labelled data is demonstrated in Fig. 4d, where the inference time in hours decreases as more samples are fully refined by the System II module. Note that we did not observe any cases where System II is no longer required (sufficient experience to reduce System II thinking time to zero), possibly due to the complexity and difficulty of the cancer localisation task.

Exploring explicit reasoning in machine System II cognition: The intermediate solutions from the refinement process in System II thinking, presented in Fig. 3, show patterns of explicit reasoning in our algorithm that mimic the ability of human System II cognition. All refinements in the competition that led to higher distribution-discriminator scores, compared to the opposing lower-scoring agent, can be visualised to draw sample-specific insights and determine where and how the refinements improved solutions. As a specific example, the first row in Fig. 3 shows that the prostate gland cancerous lesion had lower intensity values and a conspicuous pattern in the peripheral zone distinct from the surrounding healthy tissue. This was challenging to identify due to the close proximity of the lesion to the transitional zone, which also has a lower intensity compared to the peripheral zone. This may likely be the reason that the deep learning systems, without System II capabilities, deemed a significant portion of the transitional zone to be a false positive pathological region. Similar gradual local adjustments are seen in other examples, e.g., in the first sample for liver tumour segmentation, where the tumour is correctly identified in System II, despite its close proximity to the falciform ligament with a similar intensity. The correct identification of only the tumour region in System II thinking, guided by the distribution scores, along with the trajectory of refinements, demonstrates its ability to overcome such challenging ambiguities in separating anatomical and pathological voxels, using inference-time refinement.

## Discussion

The proposed dual-process framework enables performance in machine vision tasks to improve with increasing thinking time, through an iterative refinement of solutions where multiple competing solution strategies are considered before enacting the refinements - much like human thought processes. This contrasts with conventional deep learning methods, where post-training gains typically require task-specific annotations, and the thinking/ inference time is fixed. In vision tasks, even recently developed foundation models and meta-learning approaches, designed for efficient data use, cannot improve through extended inference time. By contrast, System II demonstrates significant performance gains in real-world vision tasks such as cancer detection on medical images, purely by increasing thinking time. This highlights the potential to automate many challenging tasks through inference-time compute strategies, particularly when large amounts of labelled data are expensive or impossible to obtain, e.g., in data-scare domains such as rare disease identification and galaxy classification in astronomy. With compute power becoming more readily accessible, such approaches have the potential to improve a variety of challenging real-world tasks, potentially even when large data sets are available.

We emphasise that the use of dual-process terminology serves only as a high-level conceptual analogy for separating fast feed-forward prediction from slower unbounded iterative refinement, and is not intended as an emulated cognitive model. Our empirical work shows the performance improvements, regardless of the availability of task-specific data, possible by enabling inference-time compute in vision tasks where data are scarce.

As noted above, our experiments focus on a few vision tasks, chosen because they represent clinically and scientifically important settings where labelled data are scarce and conventional deep-learning methods underperform. We do not claim to address the full breadth of visual reasoning, which spans diverse domains and task families. Instead, our results demonstrate that inference-time compute or reasoning, implemented through competitive hypothesis refinement, can reliably improve performance in settings where additional labelled data are difficult or impossible to obtain. We view these studies as exemplars of a broader principle, where extending computation at inference can serve as a powerful alternative to expanding training datasets, and may generalise to a wider range of visual tasks in future work.

Although the proposed framework demonstrated strong performance across the evaluated tasks, several potential failure modes are worth noting. First, because System II optimisation uses the discriminator score as a learned proxy for the true task objective, occasional discrepancies between the discriminator and the true metric (e.g., Dice or IoU) could lead to refinements that improve the proxy score without improving the underlying segmentation quality. Second, when the initial System I prediction is extremely poor, a larger number of refinement iterations may be required to approach an accurate solution, potentially exceeding practical inference-time compute budgets in our applications. Third, the discriminator is adapted using a small set of labelled samples from the target domain, and if this adaptation set is unrepresentative (for example, containing predominantly large lesions), performance on substantially different structures may be affected. Cross-task pre-training substantially mitigates this risk by making the reasoning process less sensitive to the particular small adaptation set used. While some performance degradation may still arise in the presence of highly atypical outlier data, this is unlikely to affect a whole adaptation set in practice, and can be further minimised through appropriate adaptation-set design and selection.

The proposed refinement process shares some similarities with iterative denoising in diffusion models, however, the underlying mechanism is fundamentally different. Diffusion models learn a fixed reverse process from large datasets that maps noisy inputs to the data distribution, and apply this process at inference. In contrast, our framework performs per-sample optimisation guided by a learned discriminator, where candidate refinements are iteratively generated, evaluated, and selected. This enables adaptive inference-time refinement conditioned on the specific sample and task objective, allowing direct per-sample optimisation rather than following a predefined denoising path.

While we model System I thinking as an adaptable neural network trained across various tasks, there is potential to consider existing foundation models in this framework and improve their predictions via the proposed inference-time refinement through System II thinking. It is also noteworthy that while our competitive refinement framework for enabling System II thinking provides a general mechanism applicable to various vision problems, some tasks may benefit from different search-based formulations or guiding inference-time computation through human feedback formulated as a reward signal.

In addition to exploring a new research direction in modelling anthropomorphic reasoning and decision-making in machines, the System II cognitive process opens a new data-efficient, performance-improving mechanism, applicable in common real-world scenarios where acquiring labelled data to improve performance may be expensive or prohibitive.

## Methods

**Fig. 5:**
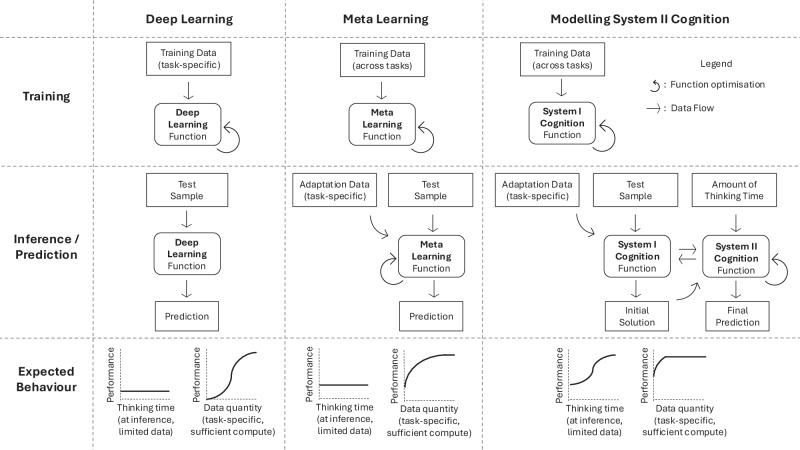
Different learning strategies are compared: deep learning for a single forward pass prediction, meta-learning for adapting to new tasks (akin to fine-tuning pre-trained foundation models), and the proposed System II cognition for allowing inference-time compute. Details of these are described in the text.

An overview of the proposed approach, along with a comparison to conventional approaches, is presented in Fig. 5.

For our method, we propose a System I module that consists of two neural networks trained adversarially^73^ across a variety of tasks, using human-labelled data. This mimics the experience-driven System I thinking in human psychology. The first network in this module is a task-predictor, which produces a solution (such as localising an object) given an input image. The second network is a distribution-discriminator which measures the likelihood of solutions being correct, by leveraging pre-learned knowledge from a variety of human-labelled data across multiple tasks. In the adversarial training, human labels are regarded as correct solutions and task-predictor solutions are regarded as incorrect. The aim during training is for the task-predictor to produce solutions close to human labels, and the distribution-discriminator to differentiate between human and task-predictor solutions. At inference for a new task, the task-predictor can be adapted to produce an initial solution for inference-time reasoning in the System II module, while the distribution-discriminator can be adapted to reward refinement of solutions towards the direction of correct solutions. This adaptation uses a small number of human-labelled samples (8–16 in our experiments) to adapt both the task-predictor and distribution-discriminator prior to System II refinement to ensure task-specific predictions for the new task. The adapted distribution-discriminator in our framework is used to guide System II refinements, as it is considered more easily generalisable than the task-predictor (with higher-dimensional outputs such as pixel-level segmentation). This offers an interesting explanation for the potential performance improvement, consistent with findings from both psychology^74^ (e.g., it is easy to recognise good writing vs bad but it is difficult to produce a well-written piece-of-work) and machine learning literature^75^ (analogous to the discriminative-vs-generative comparison, also due to the size of solution space), as well as our own experiments (see the ‘Ablation studies’ section in supplementary materials).

The System II module aims to refine the solution for a specific sample from the target task. We implement the refinement as a flexible self-play reinforcement learning^76,77^ process, where two competing neural network agents propose refinements to an initial solution produced by the task-predictor. The distribution-discriminator scores the competing refinements by measuring the likelihood of correctness for each. The refinement with a higher score is rewarded and enacted. The competing networks are then updated, using this distribution-discriminator score-based reward, to produce progressively better competing refinements in subsequent iterations. The process repeats, and the solution is iteratively refined until no further improvement is possible, as measured by the distribution-discriminator score. This iterative refinement-proposal and evaluation facilitates a dynamic improvement process where each refined solution becomes the starting point for further optimisation. This iterative self-play process thus facilitates the System II thinking module to improve solutions with added thinking time.

The final refined solution from the System II module can be used to re-train the System I module and transition tasks to fast thinking as experience grows. So, as more samples are exposed to System II thinking, the new task-specific experience of the System I module grows, which enables better initial solutions with increasing experience. This eventually reduces the time and reliance on the auto-competing System II stage. However, whether or not a task can be fully transitioned to System I, requiring no refinement in the System II stage, depends on the complexity of the task and the ability to cover task variability sufficiently. As demonstrated in the results, certain tasks may have an upper-bound of performance, which can be achieved either through extended reasoning in System II thinking or through an expansive dataset covering the task variability. Nevertheless, complex tasks may always require System II thinking to obtain optimal performance, given finite training data unable to cover task variability.

The System II framework also models the explicit reasoning in System II thinking. Due to the iterative nature of the solution-refining System II module, the full trajectory of refinement provides a record of where and how solutions were improved, enhancing interpretability and offering insights into justifications for specific final solutions.

The framework is summarised in Fig. 2a, b. Detailed methodological descriptions for implementing and training of the functions, in both modules, are presented in the ‘Methodological description’ section of the supplementary materials and open-sourced at github.com/s-sd/system-II-vision^78^, with data available from^79,80^.

## Supplementary information

Supplementary Information

Transparent Peer Review file

## Data Availability

The MNIST data used in this study were created within^78^ available at github.com/s-sd/system-II-vision. The ImageNet S data is available openly via^53^, access can be obtained by following github.com/LUSSeg/ImageNet-S. The medical imaging data is all openly available with details provided in the Supplementary Table S3; and an example data loader and minimal set provided in ref. ^78^ available at github.com/s-sd/system-II-vision.

## Code availability

The code is available openly via ^78^, available at github.com/s-sd/system-II-vision.
