# Supplementary material for: Reasoning in machine vision by learning fast and slow thinking
Source: Nat Commun. 2026 Jun 23;17:7895. doi: 10.1038/s41467-026-74579-8 (PMC13443672; doi:10.1038/s41467-026-74579-8)
Supplement: Supplementary file 1 [file 41467_2026_74579_MOESM1_ESM.pdf]

Reasoning in machine vision by learning fast and slow thinking
Supplementary Material

Shaheer U. Saeed[1,2,3,4,5*], Yipei Wang[4,5],
Veeru Kasivisvanathan[6,7], Brian R. Davidson[8],
Matthew J. Clarkson[4,5], Yipeng Hu[4,5,9], Daniel C. Alexander[4,9]

[1]Centre for Bioengineering, Queen Mary University of London, London, UK
[2]Digital Environment Research Institute, Queen Mary University of London, London, UK
[3]School of Engineering and Materials Science, Queen Mary University of London, London, UK
[4]UCL Hawkes Institute, University College London, London, UK
[5]Department of Medical Physics and Biomedical Engineering, University College London, London, UK
[6]Centre for Urology Imaging, Prostate, AI and Surgical Studies (COMPASS) Research Group, Division of Surgery and Interventional Science, University College London, London, UK
[7]Department of Urology, Comprehensive Cancer Center, Medical University of Vienna, Vienna, Austria
[8]Division of Surgery and Interventional Science, University College London, London, UK
[9]Department of Computer Science, University College London, London, UK
*Correspondence e-mail: shaheer.saeed@qmul.ac.uk

Content

Methodological description
Methodological definitions
Materials and experimental details
Supplementary results
Supplementary Figure S1 to S3
Algo. S1 to S2
Supplementary Table S1 to S9
References [1] to [34]

Methodological description

Our method emulates human dual-process cognition by integrating two distinct yet interconnected modules: the System I module, responsible for fast inference, and the System II module, designed for resource-intensive reasoning.

For the System I module, we use adversarial learning [1] to train two deep neural networks: the 'task-predictor' which predicts solutions to the task (such as segmenting a cat in an image), and the 'distribution-discriminator' which decides how likely the solution is to be 'correct'. During training, the likelihood of the solution being correct is determined by comparing task-predictor solutions to human labels. When these networks are trained across various tasks, the respective capabilities, generating solutions and predicting correctness of these solutions, can be adapted to new tasks. In general, however, the direct prediction from task-predictor performs poorly in unfamiliar or complex scenarios, and therefore resorts to the System II module to reason about and refine solutions.

The System II module not only conducts inference-time computation to refine solutions but also updates the System I module with new insights, reducing reliance on System II for similar tasks, after the one-time inference stage that is compute-intensive. This module consists of two neural networks competing against each other by iteratively hypothesising refinements to solutions (e.g., for segmenting a dog in an image, the refinement may be a change in whether certain pixels belong to the dog or not). The initial solution comes form an adapted task-predictor from the System I module, whilst the likelihood of the refinements being correct in the competing process is predicted by the adapted distribution-discriminator. The initial solution is refined until no further improvement in the likelihood of the solution being correct is possible, at which point the sample is considered 'solved'. The solved samples are used to update the System I module, which can then better and much more efficiently predict solutions for similar future tasks. This approach mimics human cognition which commits new scenarios to memory, reducing System II thinking time for future tasks. With sufficient experience, tasks may fully transition to System I, where System II thinking may no longer be required.

We present a mathematical formulation for our method in the following subsections. An overview is presented in Supplementary Figure S1.

The System I module - for adaptable initial solutions and distribution scoring

Without loss of generality assume that an image sample $x \in \mathcal{X}$ has a human label $y_h \in \mathcal{Y}_h$ (e.g., a binary image mask to delineate an object's boundaries), with $\mathcal{X}$ and $\mathcal{Y}_h$ denoting the image and human label distributions, respectively.

The **task-predictor** $f(x; w) = y_t : \mathcal{X} \rightarrow \mathcal{Y}_t$, with network weights w, aims to approximate the human label $y_h \in \mathcal{Y}_h$, where the task-predictor's output label prediction belongs to the task-predictor label distribution denoted as $y_t \in \mathcal{Y}_t$.

The **distribution-discriminator** $d(x, y; \theta) = \hat{z} : \mathcal{X} \times (\mathcal{Y}_h + \mathcal{Y}_t) \rightarrow [0, 1]$, with network weights θ, predicts a score $\hat{z} \in [0, 1]$ between 0 and 1 given an image-label pair (x, y), where $y \in \mathcal{Y}_h + \mathcal{Y}_t$ may be from either the human $\mathcal{Y}_h$ or task-predictor $\mathcal{Y}_t$ label distributions. A

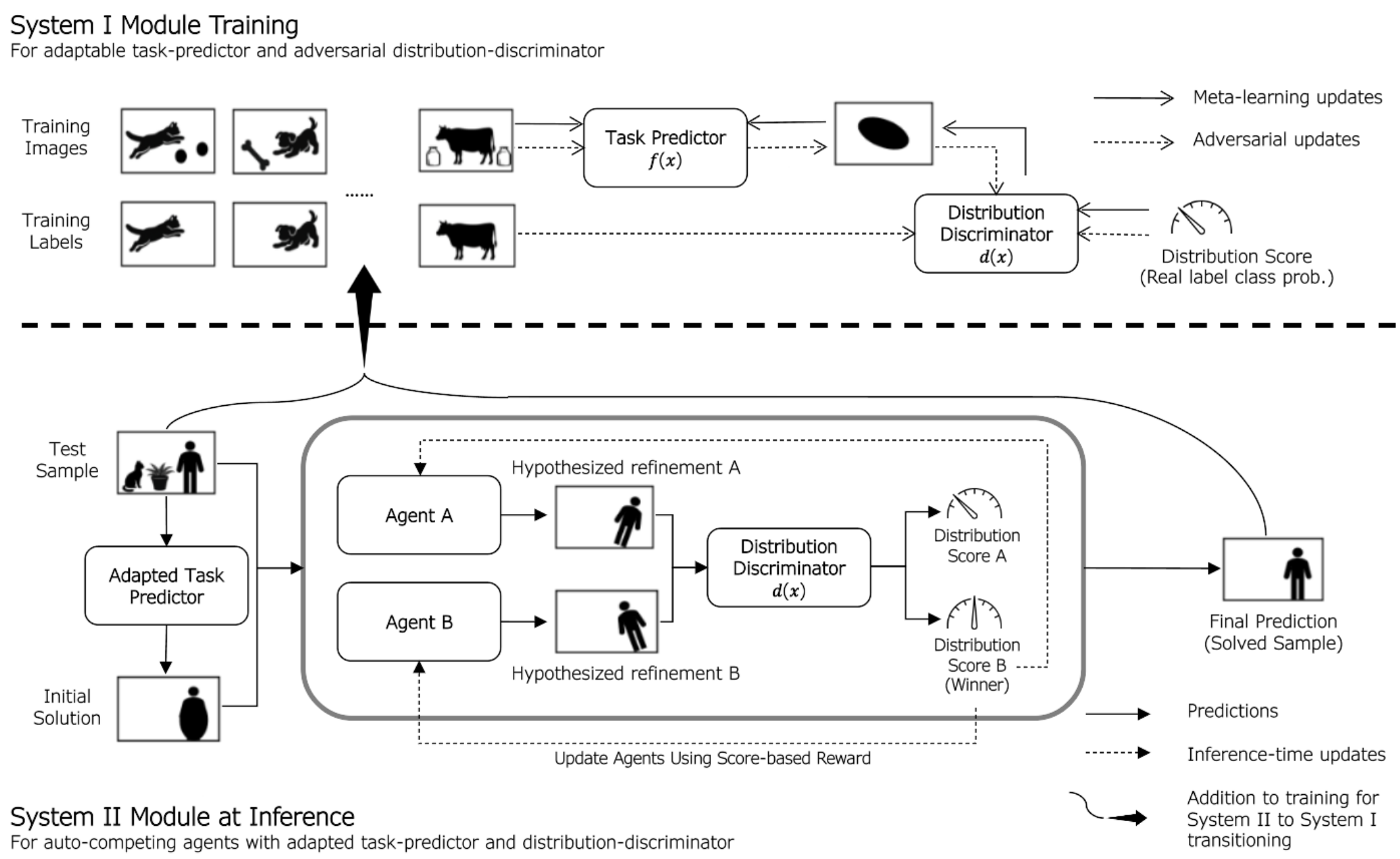

Supplementary Figure S1: An overview of the dual process approach, with the data flow and functions summarised for a target task of human segmentation on images. Note: icons are generated using DALLE-3 available via OpenAI ChatGPT under a Plus subscription.

score of zero indicates the pair being from the task-predictor label distribution $\mathcal{Y}_t$, and a score of 1 indicates the pair being from the human label distribution $\mathcal{Y}_h$, i.e. $z = 0$ if $y \in \mathcal{Y}_t$ and $z = 1$ if $y \in \mathcal{Y}_h$ where $z \in \{0, 1\}$ is its random variable representing the distinction between human and task-predictor labels.

Adversarial training is used to train the two networks. Let $\mathcal{L}(\cdot) : \mathbb{R}^1 \times \mathbb{R}^1 \to \mathbb{R}^1$ be a function that measures the difference between the distribution-discriminator prediction $\hat{z}$ and the underlying distribution score label z. The distribution-discriminator can thus learn to distinguish between task-predictor and human labels. The optimal weights of the distribution-discriminator, θ^*, can be obtained by solving the following optimisation problem, using human labels as well as task-predictor labels:

$$\theta^* = \arg\min_{\theta} \mathop{\mathbb{E}}_{x \sim \mathcal{X},\ y \sim \mathcal{Y}_h + \mathcal{Y}_t} [\mathcal{L}(z,\ \hat{z})], \quad \text{where } \hat{z} = d(x, y; \theta). \tag{1}$$

Note that the loss function for optimising Eq. 1 that measures the difference between two real numbers is defined as $\mathcal{L}(\cdot) : \mathbb{R}^1 \times \mathbb{R}^1 \to \mathbb{R}^1$, where it takes the form:

$$\mathcal{L}(a, b) = -\left[a \times \log(b)\right] - \left[(1 - a) \times \log(1 - b)\right] \tag{2}$$

The task-predictor learns to approximate human labels by minimising the difference between the distribution scores of the task-predictor $\hat{z}$ and the distribution scores for human labels (i.e., 1), by updating the task-predictor weights towards the optimal weights w^*, in the following optimisation problem:

$$w^* = \arg\min_{w} \mathop{\mathbb{E}}_{x \sim \mathcal{X}} [\mathcal{L}(1,\ \hat{z})], \quad \text{where } \hat{z} = d(x, y_t; \theta), \text{ and } y_t = f(x; w). \tag{3}$$

In a practical algorithm, the distribution- and task-predictor weight-updating steps are iterated alternatively, until equilibrium [1].

In our work, we assume that during training, the image $x \in \mathcal{X}$ is sampled from a distribution of abdominal CT or MR images, and the human-label $y \in \mathcal{Y}_h$ is a segmentation label, where segmentation tasks include various organ and region-of-interest segmentation tasks. Training across a variety of tasks [2] ensures that the task- and distribution-discriminators are generalisable to other tasks on abdominal CT or MR images, during inference. We use the Reptile algorithm [2] for meta-learning across various tasks as outlined in Algo1.

After training the System I module, this can be **adapted to new tasks** (following Eq. 1 and 2), that did not form part of the training data. This step enables the System I module to output better initial solutions (to be refined by the System II module) and more accurate

distribution-discriminator scores, for the new task. This can be achieved using a few human-labelled samples (between 4-16 in our experiments) and following the same update procedure as in training (following Eq. 1 and 2 and Algo. 1, with details on convergence outlined in the Methodological details and definitions section). Then, the adapted task-predictor predicts labels for the new task, for a new sample x_n, following $f(x_n; w^*) = y_{t,n}$. The distribution score $\hat{z} \in [0, 1]$ for this particular sample can be computed using the adapted distribution-discriminator $\hat{z} = d(x_n, y_{t,n}; \theta^*)$.

Algorithm for training and adapting the System I module: We propose to train the System I module across various tasks such that it is adaptable to any new tasks with limited labelled samples. This is achieved by employing the Reptile algorithm during training as summarised in Algo. 1. We can define sample (x^j, y^j) as coming from task j, with a total of J tasks available during training.

Data: N image-label pairs from each of J tasks $\{\{x^j_n, y^j_{h,n}\}^N_{n=1}\}^J_{j=1}$
Result: Trained distribution-discriminator $d(\cdot; \theta^*)$ and task-predictor $f(\cdot; w^*)$

```
while not converged do
    Randomly sample a task j ∈ [1, J]
    For task j, randomly sample B image-label pairs {x^j_n, y^j_{h,n}}^B_{n=1}
    for k ← 1 to K do
        Compute task-predictions f(x_n; w)^B_{n=1} = yt^B_{n=1}
        Combine human and task-predictor labels {y}^{2B}_{n=1} = {y_{h,n}}^B_{n=1} ⋃ {y_{t,n}}^B_{n=1}
        Get corresponding underlying distribution scores {z}^{2B}_{n=1} = {1}^B_{n=1} ⋃ {0}^B_{n=1}
        Assemble image samples {x}^{2B}_{n=1} = {x}^B_{n=1} ⋃ {x}^B_{n=1}
        Compute distribution-predictions ẑ^{2B}_{n=1} = d(x_n, y_n; θ)^{2B}_{n=1}
        Take one step of gradient descent to get θ_new following
          argmin_θ E_{x, y} [L(z, ẑ)]
        Update θ ← θ + ε(θ_new − θ)
        Compute new distribution-predictions ẑ^B_{n=1} = d(x_n, y_{t,n}; θ)^B_{n=1}
        Take one step of gradient descent to get w_new following argmin_w E_x [L(1, ẑ)]
        Update w ← w + ε(w_new − w)
    end
end
```

Algorithm 1: Training an adaptable System I module

The same scheme as Algo. 1 can be used to adapt the System I module to a new task, in which only the new task is sampled, instead of randomly sampling a task from J tasks.

The System II module - for an iterative competition between solution refinements

Initial solution using the System I module: The System II module aims to refine the task-predictor label $y_{t,n}$ for a new sample x_n. Since the System II module does not involve any human labels, and works on a per-sample basis, we now omit the subscripts t and n from $y_{t,n}$, in favour of the notationally convenient y, to aid readability. So before conducting System II thinking for a new sample x, we first compute an initial solution using the System I task-predictor $y = f(x; w^*)$.

Competing solution refinement functions: The auto-competing process in System II thinking involves two competing functions that iteratively refine the task-predictor label. Since the process is iterative, we use a subscript i to denote the iteration number. The competing function is denoted as $h(x, y_i; \phi) = y_{i+1} : \mathcal{X} \times \mathcal{Y}_t \to \mathcal{Y}_t$, with weights ϕ, where both the pre-refinement label y_i and post-refinement label y_{i+1} are assumed to belong to the task-predictor distribution $y_i, y_{i+1} \in \mathcal{Y}_t$, even though the actual distribution of refined labels may be different from the task-predictor label distribution. Since there are two competing functions a and b we can denote the two competitors as $h(\cdot; \phi_a)$ and $h(\cdot; \phi_b)$, with the only difference between the two being the function weights. In practice, weights ϕ_b are either a copy of ϕ_a, or ϕ_b are sampled from a distribution over all historical weights during training and the weights ϕ_a are the most up-to-date weights during training (see further implementation details in 'Methodological details and definitions' section).

Optimising the competitors during the auto-competing process: Starting with a new sample x and its task-predictor label y at iteration $i = 0$, both competitors refine the label $h(x, y_i; \phi_a) = y_{i+1,a}$ and $h(x, y_i; \phi_b) = y_{i+1,b}$; here, and in further analyses, subscripts of a or b denote outputs from competitors a or b, respectively. The distribution score is measured for both refined labels $d(x, y_{i+1,a}; \theta^*) = \hat{z}_{i,a}$ and $d(x, y_{i+1,b}; \theta^*) = \hat{z}_{i,b}$. These distribution scores are used to formulate a reward for competitor a:

$$R_i = \begin{cases} +1 & \textbf{if } \hat{z}_{i,a} \geq \hat{z}_{i,b} \\ -1 & \text{otherwise} \end{cases} \tag{4}$$

This reward is used to train both competitors and update weights ϕ towards optimal weights ϕ^*. For the next iteration $i = i+1$, the refined solution with the higher distribution-discriminator score is set as y_{i+1}, meaning that we only use the winning refinement to further refine in the next iterations. We allow iteration of this process until a maximum iteration number i_{end} (see 'Methodological details and definitions' for procedure of computing the value of i_{end}). We can then reset the refinements and repeat the iterative refinement to collect multiple sets of $[R_i]_{i=0}^{i_{end}}$. The optimisation of weights ϕ, using experience from both competing sides, can be formulated as the following optimisation problem over multiple collected sets of rewards:

$$\phi^* = \arg\max_{\phi} \left[\mathop{\mathbb{E}}_{y_{i,a} \sim h(\cdot;\phi_a)} \left[\sum_{i=0}^{i_{end}} R_i \right] + \mathop{\mathbb{E}}_{y_{i,b} \sim h(\cdot;\phi_b)} \left[\sum_{i=0}^{i_{end}} -R_i \right] \right] \tag{5}$$

Solving the above optimisation problem for a single sample x means that no further improvements in the distribution score are possible and thus the fully-refined sample is considered as solved by the System II thinking module. This reward-based training is known as reinforcement learning self-play[3,4], where competitors start out with random behaviours which are then refined towards maximising the reward. Practically it may be beneficial to aggregate updates to the competitor weights across a few different samples (up to 8 tested in our experiments), to ensure stable training[5,6,7]. As opposed to directly optimising the task-predictor using the distribution score for a single new sample e.g., using the adversarial training framework to model System II, our auto-competing process which incorporates exploration by the use of reinforcement learning, ensures that the optimisation does not get stuck in local optima, which is a common problem with other adversarial learning frameworks[1,8,9,10].

System II refinement is initialised from the prediction of the System I task-predictor to provide a reasonable starting hypothesis for the iterative optimisation process. However, the refinement objective is defined solely by the discriminator score, and therefore the reasoning process is not constrained to remain close to the initial prediction. Instead, candidate refinements are iteratively explored and selected based on their impact on the discriminator score.

For each refinement step, System II selects the single best hypothesis rather than merging the two proposals. This choice is motivated by firstly the fact that many competing refinements are mutually contradictory (e.g., expanding versus contracting a boundary), where merging would produce semantically inconsistent shapes, and secondly the use of relative, rather than absolute, discriminator-based reward assignment, which provides stability against small fluctuations in absolute discriminator scores.

Although the objective of the System II module is to improve segmentation quality, refinement is structured as pairwise competition rather than direct maximisation of the discriminator score. As the discriminator provides a learned proxy rather than the true task metric at inference, relying on absolute score optimisation can be sensitive to calibration noise. Instead, at each iteration two candidate refinements are evaluated comparatively, and only the higher-scoring proposal is enacted and rewarded. This relative reward formulation produces a stable binary reinforcement signal and reduces sensitivity to small fluctuations in the absolute discriminator outputs. Limiting the process to pairwise competition, rather than multiple simultaneous comparisons, maintains a well-conditioned optimisation signal (less impacted by absolute discriminator scores) by avoiding the need to rank or weight multiple competing refinements. Multiple comparisons would increase sensitivity to noise in the discriminator scores, requiring ranking or weighting, leading to less stable optimisation. While such extensions are possible in principle, pairwise comparison provides a more reliable optimisation strategy under a learned reward function. Although the reward is dis-

cretised, the magnitude of improvement is implicitly preserved through selection frequency: refinements that yield larger improvements are more consistently chosen across iterations, allowing their effects to accumulate over time. This common comparison-based RL formulation retains robustness to noise in the discriminator by avoiding reliance on absolute score differences, while still favouring substantially better policies through repeated selection.

Transitioning tasks from System II to System I: After a sample is solved by System II thinking, it can be used to update the System I module using the refined label in-place of the human label (following Eq. 1 and 2) to allow the next sample to get a better initial task-predictor label, to reduce the System II thinking time for the subsequent samples. In practice, similar to aggregating updates to the competitor, updates to the System I module can also be aggregated across a few (8 in our experiments) different samples.

Preventing disconnects between the discriminator scores and actual task performance: Two mechanisms ensure alignment between the discriminator and the true task objective to be maintained in the reasoning process. First, before System II reasoning begins, both the task-predictor and distribution-discriminator are adapted using a small set of human-labelled samples specific to the target domain. This ensures that predictions from both networks are task-relevant, minimising the difference in objectives between the domain-of-interest and cross-task pre-training. Furthermore, use of the meta-learning algorithm, Reptile, during pre-training, equips the System I module with the ability to become task-relevant with minimal labelled data. Once System II reasoning begins, both networks are held fixed, preventing further drift during the refinement process itself. As neither the task-predictor nor the discriminator is updated during System II reasoning, the iterative refinement process cannot introduce or amplify biases in the learned reward signal. Second, the reward is defined as the relative improvement in discriminator score between competing refinements, rather than relying on the discriminator's absolute output. Relative rewards are considerably less sensitive to calibration errors in the discriminator, because what matters is the direction of improvement, not the specific numerical value, which is a common strategy used in RL. This comparison-based formulation mitigates the impact of calibration errors in the discriminator because optimisation depends on the direction of improvement between competing refinements rather than the absolute score values. Consequently, small biases in the discriminator are unlikely to accumulate across the iterative reasoning process. Ablation studies removing the relative reward (auto-competition) are presented in Supplementary Table S5, which shows that the relative reward formulation offers improved performance. Note that we intentionally used a few samples for adaptation (e.g., 8 samples as shown in Figure 5c of the main manuscript and Supplementary Table S6). Using a larger adaptation set may further reduce the likelihood of any potential deviation between the discriminator and true segmentation quality. However, our aim was to evaluate the system under a highly sample-efficient regime and demonstrate that strong performance can be achieved even with minimal adaptation data.

Algorithm for the auto-competing process: The auto-competing process is initialised by first obtaining an initial solution from the already-trained task-predictor. This

is then refined by both competitors in the auto-competing process. The distribution scores are computed for both refined solutions and then the one leading to a higher score wins the iteration and is selected to be used for the next iteration. On the next iteration, the refined solution is then further refined by both competitors and the entire process is repeated. Once a specified number of iterations have elapsed, we can update the competitors using gradient ascent informed by the win or loss of the iteration, formulated into a reward. Resetting the refinements and repeating these updates until convergence, we eventually obtain an optimal refinement strategy that leads to the highest distribution score. Then the sample is considered as solved by the System II thinking. This process is summarised in Algo. 2.

Data: New sample image x
Result: Optimal refinement strategy $h(\cdot;\phi^*)$

Set $H = 0$

while *not converged* **do**

  Start at $i = 0$
  Sample an initial solution using the task-predictor $f(x; w^*) = y$

  Set competitor a weights to $\phi_{a,H}$: $h(\cdot; \phi_{a,H})$
  Set competitor b weights to ϕ_{b,H_s}: $h(\cdot; \phi_{b,H_s})$

  Sample initial refinement for competitor a: $y_{i=0+1,a} = h(x, y; \phi_{a,H})$
  Sample initial refinement for competitor b: $y_{i=0+1,b} = h(x, y; \phi_{b,H_s})$

  Compute competitor a distribution score: $d(x, y_{i=0+1,a}; \theta^*) = \hat{z}_{i=0+1,a}$
  Compute competitor b distribution score: $d(x, y_{i=0+1,a}; \theta^*) = \hat{z}_{i=0+1,b}$

  Compute reward R_1 using eq 4

  If: $\hat{z}_{i=0+1,a} \geq \hat{z}_{i=0+1,b}$, **Then:** Set $y_{i+1} = y_{i=0+1,a}$ **Else:** Set $y_{i=0+1} = y_{i=0+1,b}$

  for $i \leftarrow 1$ **to** i_{end} **do**

    Sample initial refinement for competitor a: $y_{i+1,a} = h(x, y_i; \phi_{a,H})$
    Sample initial refinement for competitor b: $y_{i+1,b} = h(x, y_i; \phi_{b,H_s})$

    Compute competitor a distribution score: $d(x, y_{i+1,a}; \theta^*) = \hat{z}_{i+1,a}$
    Compute competitor b distribution score: $d(x, y_{i+1,a}; \theta^*) = \hat{z}_{i+1,b}$

    Compute reward R_{i+1} using eq 4

    If: $\hat{z}_{i+1,a} \geq \hat{z}_{i+1,b}$, **Then:** Set $y_{i+1} = y_{i=0+1,a}$ **Else:** Set $y_{i=0+1} = y_{n,i=0+1,b}$

  end
  Once $R_{i=1:i_{end}}$ collected, update weights ϕ using Eq. 5
  Set $H = H + 1$

end

Algorithm 2: System II solution refinement through and auto-competing mechanism

Methodological definitions

Competitor sampling

As outlined in Algo. 2, the variable H is iterated every-time the competitor weights are updated. The weights of competitor a are set to $\phi_{a,H}$: $h(\cdot;\phi_{a,H})$, meaning that these remain as the most up-to-date weights in the optimisation process. However, the weights of competitor b are set to ϕ_{b,H_s}: $h(\cdot;\phi_{b,h})$ where in the simplest case $H_s = H$ which is commonly known as vanilla reinforcement learning self-play[4]. In our work, we use fictitious self-play to avoid the optimisation being stuck in solutions that do not improve[4], which means that $H_s \sim 1/H$, denoting the sampling of H_s from a uniform distribution of all values between 0 and H. Practically this means that competitor b is a historic version of competitor a with weights for competitor b sampled from a uniform distribution of all historic weights of competitor a.

The definition of 'refinement'

Assuming that a grey-scale image $x \in \mathbb{R}^P$ has P pixels, we can assume that the segmentation for such an image would take the form $y \in \{0,1\}^P$. The refinement of this segmentation that we have thus far summarised as $y_{i+1} = h(x, y_i; \phi)$ can then be modelled in a number of steps as follows:

$$\begin{aligned}
\text{The refinement } y_{i+1} = h(x, y_i; \phi) \text{ is broken down as follows :} \\
\text{A segmentation label is denoted as: } y_i \in \{0,1\}^P \\
\text{In actuality } h(x, y_i; \phi) = q \in [0,1]^P \text{ , values between 0 and 1;} \\
\text{For } q \in [0,1]^P \text{ set top } p \text{ values to 1 and rest to 0, to get: } q_{rounded} \in \{0,1\}^P \\
\text{Apply refinement as an element-wise logical } \textbf{XOR} \text{ operation: } y_{i+1} = y_i \ \oplus \ q_{rounded}
\end{aligned}$$

This refinement is carried out using fixed parameters in our work, which were set based on a grid search across 5 different applications. In our work, $p = 0.01$ and $i_{end} = p \times P \times 100$.

Our implementation performs refinement at the pixel level using XOR flipping to allow maximum flexibility in the evolving solution space. This binary formulation keeps the refinement action space tractable during iterative reasoning, as the agent only decides whether pixels should be included or excluded from the segmentation rather than determining continuous probability updates. This reduces optimisation complexity and enables stable reinforcement learning convergence, particularly when compute budgets are fixed. While this operates without explicit shape priors or graph/variational constraints, anatomical plausibility is implicitly enforced via the learned distribution-discriminator, which penalises unlikely or anatomically implausible refinements. This avoids imposing hard-coded continuity assumptions, which may conflict with the irregular and heterogeneous morphologies often observed in real cancer pathology. Although refinements operate on binary masks,

uncertainty information can still be inferred from the trajectory of refinements across iterations. Regions that undergo frequent modification during the reasoning process correspond to areas of higher ambiguity in the segmentation.

The refinement process operates on the full segmentation mask and does not assume a single-object setting. When multiple objects are present, the distribution-discriminator evaluates the overall segmentation quality across the entire mask. Consequently, candidate refinements that improve some regions while degrading others are compared based on their net effect on the discriminator score, and the refinement yielding the higher overall score is enacted. Because the discriminator is trained on datasets containing multiple objects during cross-task pre-training and task-specific adaptation, it naturally learns to evaluate aggregate or global segmentation quality, even in multi-object scenarios.

The definition of convergence

The convergence in the context of training the System I module is evaluated with respect to the loss $\mathcal{L}$. Let iterations in the while loop in Algo 1 be denoted as o. The loss at iteration o can thus be denoted as L_o. The convergence criterion is defined based on the change in windowed moving average across iterations:

$$1/10 \left[\sum_{v=0}^{10-1} L_{o-v} - \sum_{v=0}^{10-1} L_{o-10-v} \right] \leq 1e-5 \tag{6}$$

The System II module is considered as converged when the value of R_i stabilises. We can first define $R_{sum} = \sum_{i=1}^{i_{end}} R_i$. Since we iterate H every time $R_{i=1:i_{end}}$ is collected, we can use H as a subscript denoting the sum of rewards at iteration H as $R_{sum,\ H}$. The convergence criterion is then defined as:

$$1/10 \left[\sum_{v=0}^{10-1} R_{sum,\ H-v} - \sum_{v=0}^{10-1} R_{sum,\ H-10-v} \right] \leq 1 \tag{7}$$

Materials and experimental details

Datasets

For pre-training the System I module, we used a collection of data sets[11], as outlined in Supplementary Table S1. All included data sets are considered relevant to the abdomino-pelvic region and thus the System I learns to be adaptable to other structures or pathologies in the abdomino-pelvic region. The subsequent adaptation to the target task required 8-16 samples.

For data sets that were used for evaluation, we split the data sets, on subject level, into a ratio of 60:40 for the development and held-out sets, to ensure sufficient statistical power for validation. For training or adaptation, we randomly sampled data from the development sets. All results are reported on the held-out sets.

Dataset	Modality	Structure	Cohort Country	Samples (Held-Out)	Role
[12]	CT	Spleen	United States	41	Training
[12]	CT	Liver Vessels	United States	303	Training
[13]	CT	Gallbladder	United States	30	Training
[13]	CT	Adrenal Gland	United States	30	Training
[13]	CT	Major Vessels	United States	30	Training
[13]	CT	Stomach	United States	30	Training
[14]	CT	Kidneys	>3 countries	119	Training
[14]	CT	Bladder	>3 countries	119	Training
[15]	MR	Liver	Turkey	15	Training
[15]	MR	Kidneys	Turkey	15	Training
[15]	MR	Spleen	Turkey	15	Training
[16]	MR	Bladder	China	40	Training
[16]	MR	Gallbladder	China	40	Training
[16]	MR	Prostate	China	40	Training
[16]	MR	Major Vessels	China	40	Training
[12]	MR	Prostate	Netherlands	48	Training
[17]	mp-MR	Prostate Tumour	United Kingdom	520 (208)	Evaluation
[12]	CT	Liver Tumour	France	131 (52)	Evaluation
[12]	CT	Pancreas Tumour	United States	282 (112)	Evaluation
[12]	CT	Colon Tumour	United States	126 (50)	Evaluation
[18]	CT	Kidney Tumour	United States	489 (195)	Evaluation

Supplementary Table S1: A summary of the data sets used for training and evaluation.

Network architectures and hyper-parameters

The hyper-parameters, including the network architectures used are summarised in Supplementary Table S2.

Task-Predictor - UNet [19]	
Input Size	256×256
Batch Size B	64
Optimiser	Adam
Reptile ϵ	0.02
Distribution-discriminator - ResNet38	
Input Size	256×256
Batch Size	64
Optimiser	Adam
Competing Refinement Functions - UNet [19]	
Input Size	$P = 256 \times 256$
Batch Size	8
Optimiser	Adam
Proportion of pixels to flip per iteration p	$p = 0.01$
Terminal Iteration i_{end}	$p \times P \times 100$

Supplementary Table S2: A summary of hyper-parameter configurations used in this work.

Supplementary results

Detailed results for computer vision tasks

Digit segmentation from noisy images For the MNIST digit classification dataset we added Gaussian noise to the images, and thresholded the original clean images at an intensity of 0.5 to generate segmentation ground-truth labels. The System I module was trained to segment digits from noisy images using digits 0–5. System II thinking, implemented through our proposed framework, was then applied to segment the remaining digits 6-9 (refer to the open-source code repository for further details). For each of the unseen target digits, we used four samples to adapt the System I module prior to System II thinking.

To evaluate performance, we tested the model on 4000 samples of these digits (i.e., digits 6-9). As shown in Figure 2 in the main manuscript, we observed dramatic performance improvements (p-value= 0.0002, paired Student's t test at a significance level of $\alpha = 0.05$) compared to supervised deep learning [19] using the same training and adaptation data as our System II approach. Our proposed System II module achieved a Dice score of 97.3±2.4, compared with the deep learning models which achieved 69.7±4.3 and 97.2±2.6, when using 4 and 6000 samples from the target digits for learning, respectively. The latter is an upper-bound performance for these new tasks.

Object segmentation for new classes Out of 919 classes in the ImageNet-S large-scale unsupervised semantic segmentation (LUSS) data set [20], we reserved 40 randomly sampled classes to evaluate our approach. Ten images were randomly sampled from each of these 40 reserved classes (for the purpose of this study, only those classes are considered if they have at least 10 images available per-class). From the remaining classes, ensuring none of the reserved held-out classes were present, 2000 training images were randomly sampled, to

train the System I module across different classes, to predict the segmentation of objects. For each of the 40 reserved classes, the System I module was adapted using 4 samples and evaluation was done on the remaining 6 samples. For these $40 \times 6 = 240$ samples, we generated segmentations using our System II thinking approach.

As shown in Figure 2 in the main manuscript, our approach obtained a mean intersection over union (mIoU) of 0.691 ± 0.047 compared to the previous best-performing method[21] that obtained 0.538 ± 0.039 mIoU when using the same training and adaptation data as our System II approach, or 0.645 ± 0.046 mIoU when using the pre-training strategy as outlined in[21] (p-values = 0.0006 and 0.003, respectively).

Comparison to existing methods for cancer localisation

In Supplementary Table S3 and S4, we present detailed results for cancer detection tasks, comparing our System II approach to not only the previous best-performing specialised deep learning models but also to foundation models and widely-used benchmarking U-Nets. The amount of human-labelled data used for training of each method is investigated. Methods that have been pre-trained refer to those using other tasks for learning as summarised in Supplementary Table S1, with the Reptile algorithm[2] being used to pre-train across these different tasks. Our method shows superior performance compared to the previously best-performing methods as well as to foundation models and specialised U-Nets trained with much larger datasets (in the order of hundreds of samples), compared to our method which only uses 8-16 samples from the target task.

Method	Pre-Train	Labels	Prostate	Liver	Pancreas	Colon	Kidney
System II	Yes	8	62.4±5.1	**85.3±8.6**	60.9±9.7	**65.1±10.7**	**75.6±6.3**
System II	Yes	12	**63.0±4.8**	83.9±10.2	63.6±8.9	63.3±10.2	74.9±5.9
System II	Yes	16	62.8±5.8	84.3±9.1	**66.2±9.4**	64.7±11.0	74.1±6.2
UNet[19]	Yes	8	24.9±5.5	32.7±10.1	24.6±8.5	34.8±10.7	31.6±6.2
UNet[19]	Yes	12	28.5±5.3	40.2±11.3	31.2±9.2	32.4±11.6	34.1±5.3
UNet[19]	Yes	16	29.8±4.7	43.1±9.4	45.0±8.7	41.7±11.1	35.3±5.7
UNet[19]	Yes	>100	41.7±5.9	74.2±9.7	56.1±9.1	53.9±11.9	71.8±6.8
SUNeTR[22]	Yes	8	25.6±5.3	35.8±10.6	26.3±8.7	34.9±9.8	34.1±6.6
SUNeTR[22]	Yes	12	28.7±5.9	39.3±10.4	33.6±9.8	36.4±11.7	35.1±6.5
SUNeTR[22]	Yes	16	34.5±4.9	45.9±9.9	46.2±9.1	45.3±10.6	43.4±5.8
SUNeTR[22]	Yes	>100	41.9±5.4	76.7±10.2	57.3±8.9	52.8±11.2	70.8±5.9
SegNeXt[23]	Yes	8	23.5±4.5	31.0±11.8	25.9±9.8	32.5±11.9	31.2±6.7
SegNeXt[23]	Yes	12	29.6±5.2	41.4±9.7	32.8±8.4	38.3±11.2	35.1±6.3
SegNeXt[23]	Yes	16	33.3±5.8	44.7±10.2	48.4±9.3	42.9±11.4	47.8±6.1
SegNeXt[23]	Yes	>100	40.2±4.9	74.0±10.5	58.2±8.9	56.7±10.7	70.9±5.8
UNet[19]	No	8	18.3±5.7	20.8±8.9	20.5±9.6	28.3±11.8	30.8±6.6
UNet[19]	No	12	22.7±4.6	27.4±9.7	33.9±9.3	30.1±11.5	32.4±5.2
UNet[19]	No	16	26.8±4.9	34.7±10.5	41.4±8.5	40.9±10.3	39.3±6.3
UNet[19]	No	>100	37.6±4.9	74.5±10.4	54.0±9.9	55.2±10.2	70.2±5.6
Dv2[24]	Yes	8	38.4±4.9	37.4±9.1	37.0±8.6	36.1±10.4	38.1±6.2
Dv2[24]	Yes	12	37.9±4.3	39.3±8.6	37.2±8.9	37.0±10.5	37.8±5.1
Dv2[24]	Yes	16	38.5±5.6	41.6±9.7	38.7±9.3	39.2±11.5	40.9±5.7
Dv2[24]	Yes	>100	40.8±5.6	73.5±10.3	55.9±9.1	53.4±10.3	72.5±6.8
USeg[25]	Yes	8	27.6±5.3	-	33.2±8.2	39.1±11.9	-
USeg[25]	Yes	12	34.7±5.8	-	34.7±8.9	40.5±11.3	-
USeg[25]	Yes	16	36.0±4.7	-	40.6±8.8	42.9±10.8	-
USeg[25]	Yes	>100	42.1±5.1	-	60.7±9.5	61.5±10.1	-
nnUNet[26]	No	>100	42.3±5.6	-	-	-	-
CLIP[27]	Yes	>100	-	79.4±8.1	62.3±9.8	63.1±10.6	-
ASeg[28]	No	>100	-	-	-	-	**76.4±5.5**

Supplementary Table S3: Cancer lesion segmentation results (Dice score, as widely reported in medical imaging literature). Where we specify > 100 human labels, the full train sets from the respective tasks have been used. **First block:** results from our System II approach; **Second block:** UNet model[19] pre-trained on the same data as our System I module using the Reptile algorithm and fine-tuned using human labels for each application; **Third block:** UNet model without pre-training; **Fourth block:** foundation models (Dv2 is DINO-v2[24] encoder fine-tuned on 50% of training data and segmentation head fine-tuned on remaining data; with labelled samples for each application used to fine-tune the entire model before inference; USeg is the UniverSeg[25] model either fine-tuned or using support samples for each application - applications used in training of USeg were not considered); **Fifth block:** previous best-performing methods for each application. Where results are emboldened in more than one block, this indicates that statistical significance was not found for the comparison. Pre-train indicates the use of pre-training sets before training for the target task.

Method	Pre-Train	Labels	Prostate	Liver	Pancreas	Colon	Kidney
System II	Yes	8	45.3±5.4	**74.4±13.1**	43.8±10.0	**48.3±11.8**	**60.8±8.1**
System II	Yes	12	**46.0±5.1**	72.3±15.1	46.6±9.6	46.3±10.9	59.9±7.5
System II	Yes	16	45.8±6.2	72.9±13.6	**49.5±10.5**	47.8±12.0	58.9±7.8
UNet [19]	Yes	8	14.2±3.6	19.5±7.2	14.0±5.5	21.1±7.8	18.8±4.4
UNet [19]	Yes	12	16.6±3.6	25.2±8.9	18.5±6.5	19.3±8.3	20.6±3.9
UNet [19]	Yes	16	17.5±3.2	27.5±7.6	29.0±7.2	26.3±8.9	21.4±4.2
UNet [19]	Yes	>100	26.3±4.7	59.0±12.3	39.0±8.8	36.9±11.2	56.0±8.3
SUNeTR [22]	Yes	8	14.7±3.5	21.8±7.9	15.1±5.8	21.1±7.2	20.6±4.8
SUNeTR [22]	Yes	12	16.8±4.0	24.5±8.1	20.2±7.1	22.2±8.7	21.3±4.8
SUNeTR [22]	Yes	16	20.8±3.6	29.8±8.3	30.0±7.7	29.3±8.9	27.7±4.7
SUNeTR [22]	Yes	>100	26.5±4.3	62.2±13.4	40.2±8.7	35.9±10.3	54.8±7.1
SegNeXt [23]	Yes	8	13.3±2.9	18.3±8.3	14.9±6.5	19.4±8.5	18.5±4.7
SegNeXt [23]	Yes	12	17.4±3.6	26.1±7.7	19.6±6.0	23.7±8.6	21.3±4.6
SegNeXt [23]	Yes	16	20.0±4.2	28.8±8.5	31.9±8.1	27.3±9.2	31.4±5.3
SegNeXt [23]	Yes	>100	25.2±3.8	58.7±13.2	41.0±8.9	39.6±10.4	54.9±7.0
UNet [19]	No	8	10.1±3.5	11.6±5.5	11.4±6.0	16.5±8.0	18.2±4.6
UNet [19]	No	12	12.8±2.9	15.9±6.5	20.4±6.7	17.7±8.0	19.3±3.7
UNet [19]	No	16	15.5±3.3	21.0±7.7	26.1±6.8	25.7±8.1	24.5±4.9
UNet [19]	No	>100	23.2±3.7	59.4±13.2	37.0±9.3	38.1±9.7	54.1±6.6
Dv2 [24]	Yes	8	23.8±3.8	23.0±6.9	22.7±6.5	22.0±7.7	23.5±4.7
Dv2 [24]	Yes	12	23.4±3.3	24.5±6.7	22.9±6.7	22.7±7.9	23.3±3.9
Dv2 [24]	Yes	16	23.8±4.3	26.3±7.7	24.0±7.1	24.4±8.9	25.7±4.5
Dv2 [24]	Yes	>100	25.6±4.4	58.1±12.9	38.8±8.8	36.4±9.6	56.9±8.4
USeg [25]	Yes	8	16.0±3.6	-	19.9±5.9	24.3±9.2	-
USeg [25]	Yes	12	21.0±4.2	-	21.0±6.5	25.4±8.9	-
USeg [25]	Yes	16	22.0±3.5	-	25.5±6.9	27.3±8.8	-
USeg [25]	Yes	>100	26.7±4.1	-	43.6±9.8	44.4±10.5	-
nnUNet [26]	No	>100	26.8±4.5	-	-	-	-
CLIP [27]	Yes	>100	-	65.8±11.1	45.2±10.3	46.1±11.3	-
ASeg [28]	No	>100	-	-	-	-	**61.8±7.2**

Supplementary Table S4: The mean intersection over union (mIoU) metric reported for cancer lesion segmentation, corresponding to the above Supplementary Table S3 (where Dice was reported).

Retrospective evaluation against human experts for prostate cancer detection

We evaluate performance against uro-radiology-specialised radiologists in prostate cancer detection, in which ground-truth labels are available from independent histpathology examinations. We trained our System II cognition using 16 samples with histopathology labels derived from prostate template-mapping biopsy to indicate cancer presence in one of the twenty Barzell zones within the gland[29]. Each zone had a label of one to indicate positive cancer with a Gleason grade $\geq$ 3+4, and zero otherwise. We observed an across-zone sensitivity and specificity of 0.774 and 0.582. From the zonal predictions, we predicted patient-wise classification (by predicting cancer presence when any single zone was deemed cancer-positive). We used zonal prediction to compute patient-wise classification, as zonal prediction can be formulated as low-resolution segmentation and thus does not require retraining of the System I module for classification tasks, in addition to its potential spatial interpretability.

Ablation and robustness studies

For the task of liver tumour segmentation, we also present ablation studies in Supplementary Table S5, where we remove components including adversarial training and RL from our framework to evaluate their impact on task performance. When adversarial training is omitted, the two networks in the System I module are trained separately, where the task-predictor is first trained to perform the task and then a distribution-discriminator is separately trained after training the task-predictor. When auto-competition is omitted, this refers to using a single agent RL framework with a reward based on distribution-discriminator score, which is directly maximised, as opposed to difference in scores between the predictions from two competing agents. Where RL is omitted, the task-predictor is optimised directly per-sample by maximising the distribution-discriminator score as in the adversarial learning for each new sample. The results show that all of these components contribute positively towards performance.

In particular, it is interesting to note that the removal of adversarial training led to a marked reduction in performance, which highlights the interdependent nature of the solution prediction and distribution discrimination. During adversarial training, the discriminator continuously adapts to the evolving predictor outputs, while the predictor updates to better match the learned solution distribution. This coupling enables System I to capture the interdependence between solution quality and distributional plausibility, which is lost when the components are trained separately as conventional neural networks.

In addition to these ablations, we also study the impact of different hyper-parameters and amounts of labelled data, as summarised below.

Amounts of human-labelled data for adaptation: To analyse the alignment between task performance and the discriminator score, we investigate the correlation between Dice score and the discriminator score, for the task of prostate cancer segmentation, when using different amounts of human labelled data for adaptation of the System I module. The results are presented in Supplementary Table S6 below, where we measure the Pear-

System I module			System II module		
Adversarial Training	Reptile	Pre-Training	Auto-competition	RL	Performance
✓	✓	✓	✓	✓	**85.3±4.3**
✓	✗	✗	✓	✓	57.9±5.1
✗	✓	✓	✓	✓	75.7±5.8
✓	✗	✓	✓	✓	**83.7±4.7**
✗	✗	✗	✓	✓	53.1±4.5
✓	✓	✓	✗	✓	78.5±6.3
✓	✓	✓	✗	✗	64.6±4.6
✓	✗	✗	✗	✗	38.7±4.9

Supplementary Table S5: Ablation study - removing components from the framework to test efficacy of different approaches. The standard deviation is reported to measure variance across 4 runs with different seeds.

son's correlation coefficient (and standard error) between the Dice score (measured between task-predictor labels, from a task-predictor adapted with 32 samples, and human labels) and discriminator score, across 100 human-labelled samples in the holdout set. We observe that approximately 8 samples allow for correlations approaching the level of alignment observed for much larger sets of labelled data. Similar behaviour was observed across all evaluated tasks. When using eight adaptation samples, the Pearson correlation between discriminator score and Dice performance was >0.78 for all evaluated tasks, indicating that the learned reward signal remained consistently aligned with the true task objective across domains.

Human labels	Correlation
0	0.21 ± 0.09
4	0.53 ± 0.09
8	0.81 ± 0.06
12	0.83 ± 0.06
16	0.79 ± 0.06
20	0.84 ± 0.05
24	0.83 ± 0.06
28	0.81 ± 0.06
32	0.80 ± 0.06

Supplementary Table S6: Impact of human-labelled data for adaptation of the System I module on the alignment between the distribution discriminator scores and actual performance (Dice).

Impact of annotation noise: We study the impact of noisy human annotated data on the performance of our proposed method compared to deep learning. The detailed quantitative results are presented in Supplementary Table S7. Note that we added random deformations to the segmentation labels to simulate human error in annotation, the biggest source of noise in our data. The segmentation masks were deformed using a grid of 3×3 control points, each

with a random displacement of 0-40 pixels or 0-20mm, with dense displacements computed using linear interpolation. The images themselves were kept unchanged, as they already contain real-world noise sources (e.g., motion artefacts, probe variability and sensor noise), and because realistic simulation of imaging noise remains an open research problem without standardised models. Additionally, For different proportions of noisy adaptation samples, we compared the proposed method with a deep-learning-based segmentation baseline. As shown below, both methods experience performance degradation as noise increases, but our approach shows a more gradual decline, indicating better robustness to annotation outliers.

Percentage corrupted	System II	DL - Pre-trained
0	62.7±5.7	40.6±4.9
25	60.1±5.2	31.4±5.9
50	51.3±5.9	26.8±5.6
75	40.2±5.4	22.6±4.8
100	30.2±5.2	21.3±5.5

Supplementary Table S7: Studying the impact of noisy data on performance for the prostate cancer segmentation task. The 'percentage corrupted' refers to the percentage of adaptation data to which noise was added out of a total of 32 adaptation samples.

Impact of compute on per-sample performance: Supplementary Figure S2 shows the performance gain per-sample against the number of iterations. We observe an overall reduction in gain, towards zero, with an increasing number of iterations. This highlights that the proposed auto-competing mechanism converges to stable values as the refinement in the System II module progresses.

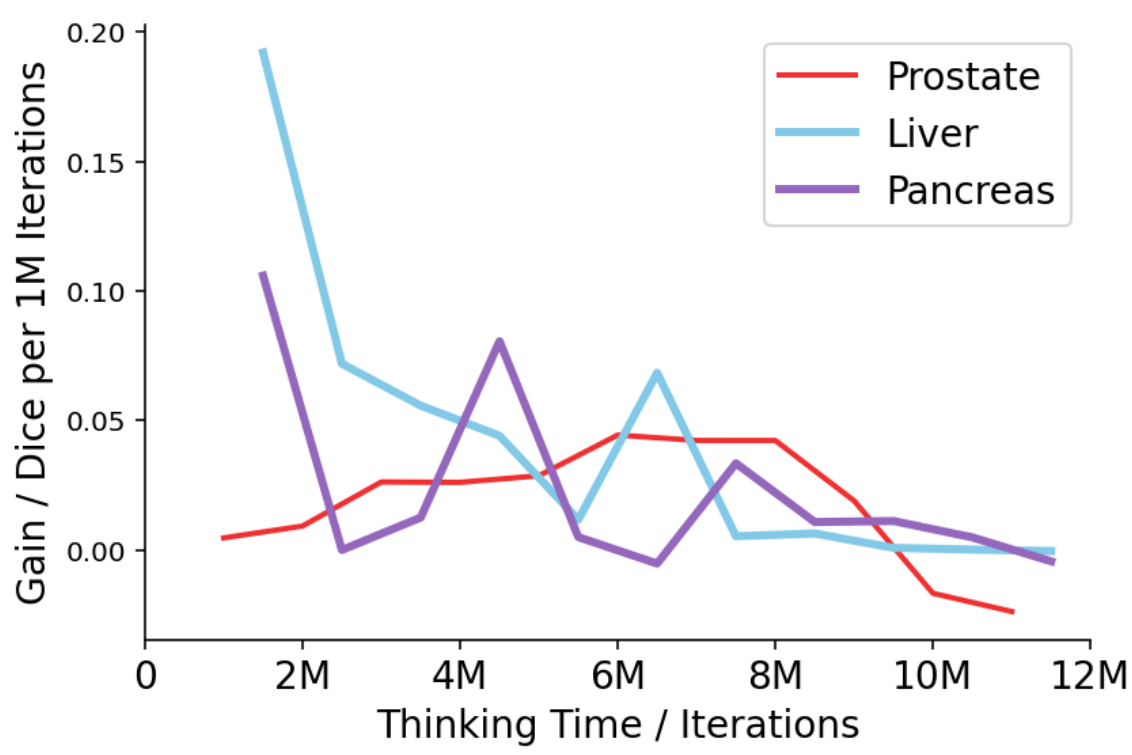

Supplementary Figure S2: Performance gain per-sample against number of compute iterations

Analysis of hyper-parameters: The analysis of hyper-parameters p (the proportion of pixels to flip per iteration) and i_{end} is presented in Supplementary Table S8. Note that we observed a high dependence of the optimal values for both hyper-parameters on the input size or number of pixels in the image P. As such, we defined i_{end} as a function of P. As the

input size stays consistent across datasets in our work, we observed a consistent optimal value for both p and i_{end} across the evaluation datasets. However, for other datasets, in future work, these parameters may need to be tuned according to the input or ROI size.

Proportion p	Termination i_{end}	Prostate	Liver	Pancreas
0.001	$p \times P \times 1$	56.8±5.7	74.3±8.8	-
0.005	$p \times P \times 1$	60.4±4.9	79.1±9.2	62.9±8.8
0.010	$p \times P \times 1$	61.7±5.8	81.5±9.7	65.2±9.2
0.050	$p \times P \times 1$	61.3±6.3	78.7±9.1	62.4±9.9
0.100	$p \times P \times 1$	50.4±7.8	65.3±11.7	-
0.010	$p \times P \times 1$	61.7±5.8	81.5±9.7	-
0.010	$p \times P \times 10$	60.6±5.4	83.7±9.5	65.6±9.1
0.010	$p \times P \times 100$	63.0±4.8	85.3±8.6	66.2±9.4
0.010	$p \times P \times 1000$	58.4±5.9	80.5±9.5	61.1±8.7
0.010	$p \times P \times 10000$	54.1±6.7	78.4±9.4	-

Supplementary Table S8: Impact of hyper-parameters on performance for models adapted using 8 labelled samples.

Uncertainty quantification with respect to data

To quantify the uncertainty in the computed segmentation using our proposed approach, we reserved a set of 32 samples from the liver tumour segmentation dataset. To each of these samples and their ground-truth human expert labels, we applied 32 random affine transformations. The transformations altered translation, scale, shear, rotation or a combination of these corresponding to application-specific transformations that may be observed due to shifts in imaging camera positions, variations in patient positions and variations in anatomical appearances due to noise from processes such as breathing. The standard deviation in performance (Dice score) across these affine transforms is computed per-sample and then averaged across the 32 reserved samples to obtain an estimate of the aleatoric uncertainty with respect to variations in input data.

Supplementary Figure S3 present plots of these measures of uncertainty for our proposed System II machine cognition as well as other commonly used methods for segmentation. All methods show a trend of decreasing uncertainty with an increase in the number of human labelled samples used, however, our proposed System II method shows the most stable measure of uncertainty which demonstrates its reduced reliance on human labels compared to other methods.

Computational cost

We summarise the carbon footprint of training and evaluating our proposed System II machine cognition framework in Supplementary Table S9. For computing the total power consumption or kg of CO_2 produced, the given measures can be multiplied by the GPU hours.

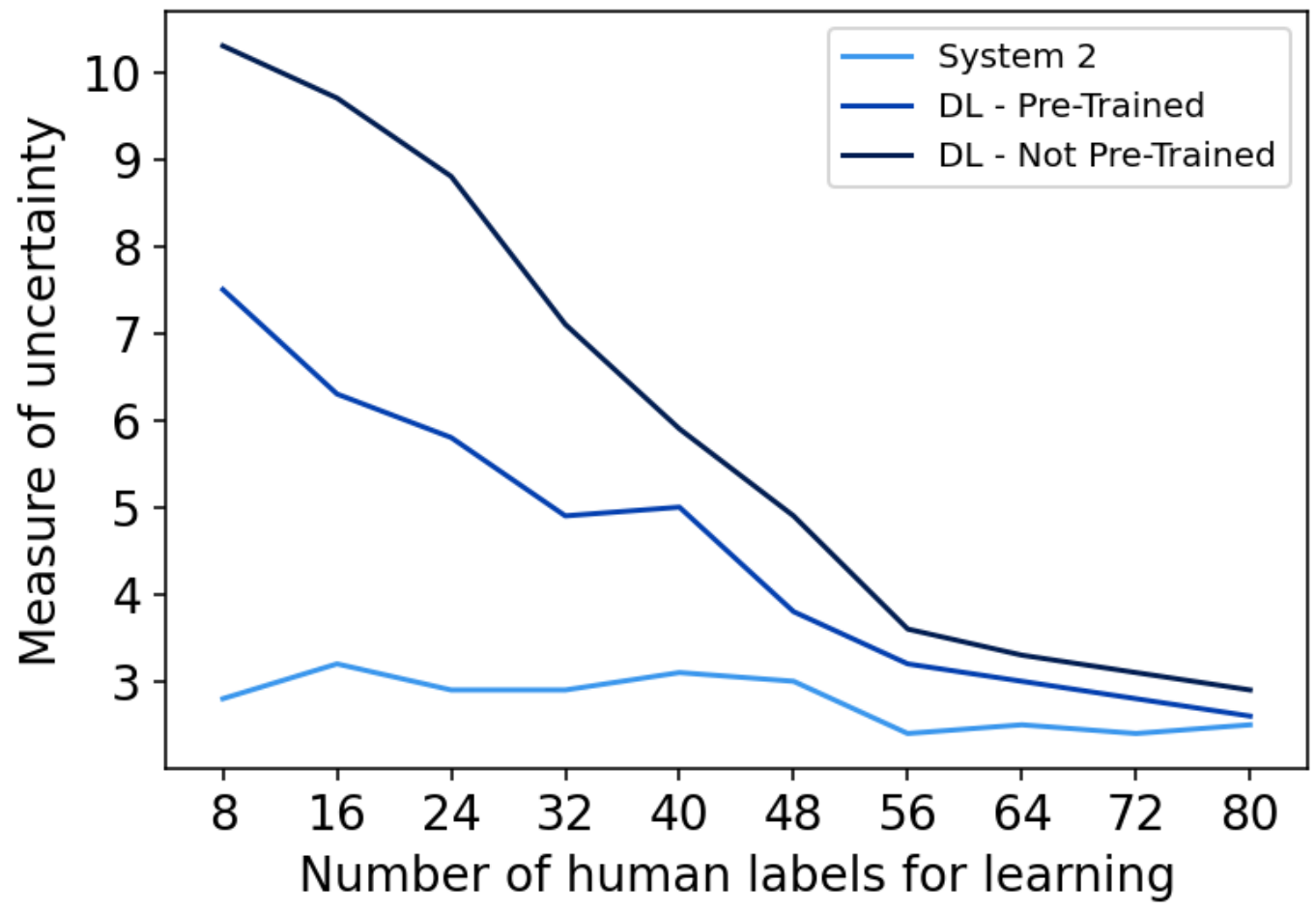

Supplementary Figure S3: Visualising uncertainty against number of human labelled samples used for learning, from the proposed and compared methods, in the liver tumour segmentation task.

Task	GPU Hours								Power	Carbon
	Pre-Train	Adapt	Inference / Reasoning Time						(W)	(kg CO2 /
			Experience (Number of Samples)							GPU hour)
			0	8	16	24	32	40		
Prostate	1152	16	41	36	24	16	6	6	300 W	0.13
Liver	1152	18	37	34	20	8	8	7	300 W	0.13
Pancreas	1152	13	42	39	19	9	7	8	300 W	0.13
Colon	1152	15	33	30	11	3	4	3	300 W	0.13
Kidney	1152	21	29	23	17	10	4	2	300 W	0.13

Supplementary Table S9: Carbon footprints estimated on Nvidia V100 GPUs.

Adapting the method for classification or regression

Our System II method can readily be adapted to other machine learning tasks by modifying the definition of the solution space and its refinement.

Classification: For classification, assume that labels are standardised to the continuous range $y \in \{0, 1\}$, representing class probabilities, with 1 being a 100% probability of belonging to the class-of-interest. For multi-class classification, we can reformulate the problem to multiple binary classification problems (classifying whether a sample belongs to class-of-interest or not) similar to[4]. To enable continuous values as labels, we apply mixup[30,31] to training images and their labels, mixing samples from the class-of-interest with out-of-class samples. The formula for mixing is $(\lambda)z_i + (1 - \lambda)z_j$, where z_i and z_j are the class-of-interest sample and out-of-class sample respectively, and λ is a mixing factor randomly sampled be-

tween 0-1. The mixing is applied to random class-of-interest and out-of-class samples to generate continuous labels for training. Once the System I module is trained with this mixed-up data, the task-predictor learns to output continuous values $f(x; w) = y \in \{0, 1\}$ for each image sample.

During System II thinking, the competing function $h(x, y_i; \phi)$ is modified to output values in the range $\{-0.01, +0.01\}$ (instead of the flipping procedure described in the definition of refinement), which can then be added to the label y_i in order to obtain y_{i+1}. Note that y_0 is initialised as 0.5, which represents a 50% probability of the sample belonging to the class-of-interest. Then during System II thinking the competing functions compete to refine the class probability for the sample, which is especially useful for ambiguous samples, to assign a representative class probability.

MNIST digit classification: We utilised the same training data as the MNIST segmentation problem discussed in the preliminary results section. This data consisted of 4000 samples from each of digits 0-9, with artificial Gaussian noise added to the images. Data from digits 0-5 was used for training the System I module for digit classification. From digits 6-9, 4 samples each were used to adapt the task predictor and distribution-discriminator prior to System II thinking. For each digit, during training and inference, due to the task binarisation process, the task was split into correct digit vs incorrect digit as opposed to a direct multi-class classification (i.e., the tasks were digit 6 vs not 6, digit 7 vs not 7, digit 8 vs not 8, and digit 9 vs not 9, instead of the multi-class representation digit 6 vs 7 vs 8 vs 9). Performing System II refinement, we observed an accuracy of 0.953, compared to 0.829 from the alternative supervised deep learning models using the same training data. The results are summarised in Supplementary Table S10.

IQ test puzzle classification This task is used to demonstrate spatial reasoning. We used the MMIQ[32] dataset for this experiment. The task predictor is configured to intake a 5-channel image with the first channel being the input puzzle (with a missing square) and the other channels being possible solution options A, B, C or D inserted into the missing square to build a completed puzzle. The System I module is trained with IQ puzzles spanning geometric transformations, spatial relationships, and logical patterns with 395, 102 and 335 puzzles in each category. The multi-class classification problem is therefore to pick the correct solution out of the 4 presented options, where this multi-class task is binarised in the same way as for the MNIST experiment.

Inference is performed on unseen movement puzzles, examples requiring pattern continuation in space. The System I module is adapted using 4 samples from these movement puzzles prior to System II applied to 379 puzzles, across which results are reported. The results, exceeding performance compared to state-of-the-art systems such as ChatGPT are presented in Supplementary Table S10 and they highlight that language based reasoning developed in multimodal systems does not readily extend to visual tasks.

Regression: Regression follows a similar formulation whereby labels are standardised to the continuous range $y \in \{0,1\}^N$, representing target values for a multiple regression problem. The System I module can be trained with these target values and learns to predict $f(x;w) = y \in \{0,1\}^N$.

Similar to classification, during System II thinking, the competing function $h(x, y_i; \phi)$ is modified to output values in the range $\{-0.01, +0.01\}$, which can be added to the label y_i in order to obtain y_{i+1}. Similarly, y_0 is initialised as 0.5. Then during System II thinking the competing functions compete to refine the target values, to reflect correct targets.

Depth estimation regression As an example of scene understanding, we evaluate the framework for depth estimation. We used the NYU Depth V2 dataset for depth estimation in natural RGB images[33]. The target values are the pixel-wise depths for the images and thus the target shape corresponds to the image shape $N = 640 \times 480$. The depths were normalised to range 0-1.

The System I module was trained with data from 20 scenes (e.g., bedroom, kitchen, bookstore, basement etc.). It was adapted to the scene 'bathroom' using 4 samples. System II refinement was performed for 64 samples, on which the results are reported in Supplementary Table S10. We demonstrate superior performance to conventional approaches given the same amount of training data.

Method	MNIST classification	IQ classification	Depth regression
	Accuracy	Accuracy	RMSE
CNN (same data)	0.829	0.244	0.514
MLP (same data)	0.746	0.239	0.535
CNN (full data)	0.931	0.271	0.379
MLP (full data)	0.927	0.261	0.443
ChatGPT5 (4-shot prompt)	-	0.264	-
Gemini2.5pro (4-shot prompt)	-	0.278	-
PixelFormer[34] (same data)	-	-	0.412
PixelFormer[34] (full data)	-	-	0.322
Ours	0.953	0.512	0.322

Supplementary Table S10: Results for classification and regression tasks using the proposed framework. 'Same data' refers to compared methods that use the same training and adaptation data as the proposed method, whereas 'full data' refers to the use of the entire dataset for training for the compared methods.

References

1. Goodfellow, I., Pouget-Abadie, J., Mirza, M., Xu, B., Warde-Farley, D., Ozair, S., Courville, A., Bengio, Y.: Generative adversarial networks. Communications of the ACM **63**(11), 139–144 (2020)

2. Nichol, A., Achiam, J., Schulman, J.: On first-order meta-learning algorithms. arXiv preprint arXiv:1803.02999 (2018)

3. Silver, D., Hubert, T., Schrittwieser, J., Antonoglou, I., Lai, M., Guez, A., Lanctot, M., Sifre, L., Kumaran, D., Graepel, T., *et al.*: A general reinforcement learning algorithm that masters chess, shogi, and go through self-play. Science **362**(6419), 1140–1144 (2018)

4. Saeed, S.U., Huang, S., Ramalhinho, J., Gayo, I.J., Montana-Brown, N., Bonmati, E., Pereira, S.P., Davidson, B., Barratt, D.C., Clarkson, M.J., et al.: Competing for pixels: a self-play algorithm for weakly-supervised semantic segmentation. IEEE Transactions on Pattern Analysis and Machine Intelligence (2024)

5. Liang, E., Liaw, R., Nishihara, R., Moritz, P., Fox, R., Goldberg, K., Gonzalez, J., Jordan, M., Stoica, I.: Rllib: Abstractions for distributed reinforcement learning. In: International Conference on Machine Learning, pp. 3053–3062 (2018). PMLR

6. Nair, A., Srinivasan, P., Blackwell, S., Alcicek, C., Fearon, R., De Maria, A., Panneershelvam, V., Suleyman, M., Beattie, C., Petersen, S., et al.: Massively parallel methods for deep reinforcement learning. arXiv preprint arXiv:1507.04296 (2015)

7. Kwok, J., Lohstroh, M., Lee, E.A.: Efficient parallel reinforcement learning framework using the reactor model. In: Proceedings of the 36th ACM Symposium on Parallelism in Algorithms and Architectures, pp. 41–51 (2024)

8. Creswell, A., White, T., Dumoulin, V., Arulkumaran, K., Sengupta, B., Bharath, A.A.: Generative adversarial networks: An overview. IEEE signal processing magazine **35**(1), 53–65 (2018)

9. Pan, Z., Yu, W., Yi, X., Khan, A., Yuan, F., Zheng, Y.: Recent progress on generative adversarial networks (gans): A survey. IEEE access **7**, 36322–36333 (2019)

10. Saxena, D., Cao, J.: Generative adversarial networks (gans) challenges, solutions, and future directions. ACM Computing Surveys (CSUR) **54**(3), 1–42 (2021)

11. Saeed, S.U., Ramalhinho, J., Pinnock, M., Shen, Z., Fu, Y., Montaña-Brown, N., Bonmati, E., Barratt, D.C., Pereira, S.P., Davidson, B., *et al.*: Active learning using adaptable task-based prioritisation. Medical Image Analysis **95**, 103181 (2024)

12. Antonelli, M., Reinke, A., Bakas, S., Farahani, K., AnnetteKopp-Schneider, Landman, B.A., Litjens, G., Menze, B., Ronneberger, O., Summers, R.M., Ginneken, B., Bilello, M., Bilic, P., Christ, P.F., Do, R.K.G., Gollub, M.J., Heckers, S.H., Huisman, H., Jarnagin, W.R., McHugo, M.K., Napel, S., Pernicka, J.S.G., Rhode, K., Tobon-Gomez, C., Vorontsov, E., Huisman, H., Meakin, J.A., Ourselin, S., Wiesenfarth, M., Arbelaez, P., Bae, B., Chen, S., Daza, L., Feng, J., He, B., Isensee, F., Ji, Y., Jia, F., Kim, N., Kim, I., Merhof, D., Pai, A., Park, B., Perslev, M., Rezaiifar, R., Rippel, O., Sarasua,

I., Shen, W., Son, J., Wachinger, C., Wang, L., Wang, Y., Xia, Y., Xu, D., Xu, Z., Zheng, Y., Simpson, A.L., Maier-Hein, L., Cardoso, M.J.: The Medical Segmentation Decathlon. arXiv (2021)

13. Synapse: Multi-Atlas Labeling Beyond the Cranial Vault - Workshop and Challenge. Online; accessed Jun 2022 (2013). `https://doi.org/10.7303/syn3193805` . `https://www.synapse.org/#!Synapse:syn3193805/wiki/217789`

14. Rister, B., Yi, D., Shivakumar, K., Nobashi, T., Rubin, D.L.: Ct-org, a new dataset for multiple organ segmentation in computed tomography. Scientific Data **7**(1), 1–9 (2020)

15. Kavur, A.E., Gezer, N.S., Barış, M., Aslan, S., Conze, P.-H., Groza, V., Pham, D.D., Chatterjee, S., Ernst, P., Özkan, S., *et al.*: Chaos challenge-combined (ct-mr) healthy abdominal organ segmentation. Medical Image Analysis **69**, 101950 (2021)

16. Ji, Y., Bai, H., Ge, C., Yang, J., Zhu, Y., Zhang, R., Li, Z., Zhanng, L., Ma, W., Wan, X., *et al.*: Amos: A large-scale abdominal multi-organ benchmark for versatile medical image segmentation. Advances in neural information processing systems **35**, 36722–36732 (2022)

17. Ahmed, H.U., Bosaily, A.E.-S., Brown, L.C., Gabe, R., Kaplan, R., Parmar, M.K., Collaco-Moraes, Y., Ward, K., Hindley, R.G., Freeman, A., *et al.*: Diagnostic accuracy of multi-parametric mri and trus biopsy in prostate cancer (promis): a paired validating confirmatory study. The Lancet **389**(10071), 815–822 (2017)

18. Heller, N., Isensee, F., Maier-Hein, K.H., Hou, X., Xie, C., Li, F., Nan, Y., Mu, G., Lin, Z., Han, M., *et al.*: The state of the art in kidney and kidney tumor segmentation in contrast-enhanced ct imaging: Results of the kits19 challenge. Medical image analysis **67**, 101821 (2021)

19. Ronneberger, O., Fischer, P., Brox, T.: U-net: Convolutional networks for biomedical image segmentation. In: Medical Image Computing and Computer-assisted intervention–MICCAI 2015: 18th International Conference, Munich, Germany, October 5-9, 2015, Proceedings, Part III 18, pp. 234–241 (2015). Springer

20. Gao, S., Li, Z.-Y., Yang, M.-H., Cheng, M.-M., Han, J., Torr, P.: Large-scale unsupervised semantic segmentation. IEEE transactions on pattern analysis and machine intelligence **45**(6), 7457–7476 (2022)

21. Gao, S., Zhou, P., Cheng, M.-M., Yan, S.: Towards sustainable self-supervised learning. arXiv preprint arXiv:2210.11016 (2022)

22. Hatamizadeh, A., Nath, V., Tang, Y., Yang, D., Roth, H.R., Xu, D.: Swin unetr: Swin transformers for semantic segmentation of brain tumors in mri images. In: International MICCAI Brainlesion Workshop, pp. 272–284 (2021). Springer

23. Guo, M.-H., Lu, C.-Z., Hou, Q., Liu, Z., Cheng, M.-M., Hu, S.-M.: Segnext: Rethinking convolutional attention design for semantic segmentation. Advances in neural information processing systems **35**, 1140–1156 (2022)

24. Oquab, M., Darcet, T., Moutakanni, T., Vo, H., Szafraniec, M., Khalidov, V., Fernandez, P., Haziza, D., Massa, F., El-Nouby, A., et al.: Dinov2: Learning robust visual features without supervision. arXiv preprint arXiv:2304.07193 (2023)

25. Butoi, V.I., Ortiz, J.J.G., Ma, T., Sabuncu, M.R., Guttag, J., Dalca, A.V.: Universeg: Universal medical image segmentation. In: Proceedings of the IEEE/CVF International Conference on Computer Vision, pp. 21438–21451 (2023)

26. Isensee, F., Jaeger, P.F., Kohl, S.A., Petersen, J., Maier-Hein, K.H.: nnu-net: a self-configuring method for deep learning-based biomedical image segmentation. Nature methods **18**(2), 203–211 (2021)

27. Liu, J., Zhang, Y., Chen, J.-N., Xiao, J., Lu, Y., A Landman, B., Yuan, Y., Yuille, A., Tang, Y., Zhou, Z.: Clip-driven universal model for organ segmentation and tumor detection. In: Proceedings of the IEEE/CVF International Conference on Computer Vision, pp. 21152–21164 (2023)

28. Myronenko, A., Yang, D., He, Y., Xu, D.: Automated 3d segmentation of kidneys and tumors in miccai kits 2023 challenge. In: International Challenge on Kidney and Kidney Tumor Segmentation, pp. 1–7. (2023)

29. Ahmed, H.U., Bosaily, A.E.-S., Brown, L.C., Gabe, R., Kaplan, R., Parmar, M.K., Collaco-Moraes, Y., Ward, K., Hindley, R.G., Freeman, A., *et al.*: Diagnostic accuracy of multi-parametric mri and trus biopsy in prostate cancer (promis): a paired validating confirmatory study. The Lancet **389**(10071), 815–822 (2017)

30. Liang, D., Yang, F., Zhang, T., Yang, P.: Understanding mixup training methods. IEEE access **6**, 58774–58783 (2018)

31. Zhang, H.: mixup: Beyond empirical risk minimization. arXiv preprint arXiv:1710.09412 (2017)

32. Cai, H., Yang, Y., Hu, W.: Mm-iq: Benchmarking human-like abstraction and reasoning in multimodal models. arXiv preprint arXiv:2502.00698 (2025)

33. Silberman, N., Hoiem, D., Kohli, P., Fergus, R.: Indoor segmentation and support inference from rgbd images. In: European Conference on Computer Vision, pp. 746–760 (2012). Springer

34. Agarwal, A., Arora, C.: Attention attention everywhere: Monocular depth prediction with skip attention. In: Proceedings of the IEEE/CVF Winter Conference on Applications of Computer Vision, pp. 5861–5870 (2023)
