# Supplementary material for: Reasoning in machine vision by learning fast and slow thinking
Source: Nat Commun. 2026 Jun 23;17:7895. doi: 10.1038/s41467-026-74579-8 (PMC13443672; doi:10.1038/s41467-026-74579-8)
Supplement: Supplementary file 2 [file 41467_2026_74579_MOESM2_ESM.pdf]

nature portfolio

Peer Review File

Reasoning in machine vision by learning fast and slow thinking

Corresponding Author: Dr Shaheer Saeed

This file contains all reviewer reports in order by version, followed by all author rebuttals in order by version.

Version 0:

Reviewer comments:

Reviewer #1

(Remarks to the Author)
The paper introduces a dual-process learning paradigm for machine vision, inspired by psychological theories of human cognition. Specifically, it proposes integrating a fast, experience-driven “System I” module with a slow, deliberative “System II” module that allows for iterative solution refinement through self-play reinforcement learning. This approach enables performance improvements with increased inference-time compute, particularly in data-scarce scenarios. Experimental results demonstrate that the proposed method outperforms conventional supervised learning, foundation models, and even human experts on standard computer vision benchmarks and cancer localization tasks across five organs. The framework shows significant promise for non-verbal machine reasoning, especially in real-world vision applications where labeled data is limited.

Strength:
+ This paper made sufficient experimental designs for the proposed method, although with some exceptions below.
+ The proposed method is effective, and can be potentially used in different applications.

Weakness:
- The System II of the method proposed in this paper relies on the probability output of the distribution-discriminator as the sole reward. Essentially, it is merely a discriminator to produce “manual annotation”, rather than a direct estimation of the actual task loss (Dice, IoU, etc.). As the mask is constantly modified during the reasoning process, the discriminator may be disconnected from the true segmentation quality. This paper does not provide a mechanism to prevent such deviations or drifts.
- I am very interested in the adaptation of the method proposed in this paper in classification. However, this paper only briefly mentions it in the appendix. Please provide more detailed experimental information and discussions for this part.
- Why does System I use the model of the distribution-discriminator architecture? This architecture is particularly poor at handling small sample situations, and the poor fine-tuning results of small samples might be caused by this reason. Would the effect be much better if it were replaced with a general neural network for segmentation?
- The refinement in the method proposed in this paper is calculated through XOR point flipping, which contradicts the anatomical continuity of actual medical organs or lesions and is prone to generating noisy shapes. The paper does not compare the refinement based on shape priors, graph optimization or variational methods.
- When comparing the segmentation performance, why do the authors choose U-Net instead of the lighter or multi-scale Swin-UNETR/SegNeXt as baseline? Compared with these methods, Unet will significantly increase the calculation without bringing quantifiable benefits.
- The System II proposed in this paper merely uses the winning refinement to further refine in the next generation. Why not consider fusing the two refinements? After all, the two refinements may have their own advantages in different segmented regions.
- The hard-coded hyperparameters such as p and i_{end} in the method of this paper are obtained through empirical grid search. Why not conduct adaptive design according to the task?
- This paper presents the computational cost of the prostate tumor segmentation task in Table 5. Please provide the computational costs of Liver, Pancreas, Colon, Kidney and other tasks involved in this paper. In addition, it is suggested to give a comparison of the reasoning delay time of different methods.
- This paper merely presents the selection of hyperparameters. It is suggested that the analysis data of hyperparameters be provided.

- Only Dice is regarded as the segmentation evaluation metric of medical images in this paper. Please appropriately add one or two evaluation metrics, such as mIoU and HF95.
- The foundation models compared in this paper are relatively outdated. It is suggested to add comparisons with the latest achievements such as SAM-Med and MedKLIP-Seg.
- In this paper, when pre-training System I, the types of medical images involved are only CT and MR. It is suggested to add images in more modalities (such as MRI).
- It is suggested that the writing of the entire paper be reorganized. The information in the main text and the appendix is scattered, and the key implementation details need to be reorganized in multiple places to be pieced together, reducing readability and reproducibility.
- Please test the robustness of the proposed method against noisy data or outliers。

(Remarks on code availability)
The code appears to be of reasonable quality and clarity for academic research purposes.

Reviewer #2

(Remarks to the Author)
Topic Summary and Overall Evaluation:

In this work, the authors present a novel framework that leverages insights and methods adapted from the literatures of inference-time adaptive computation, meta-learning, self-bootstrapping, and multi-agent (self-play) reinforcement learning to facilitate improved performance on machine vision tasks in sparse data regimes. The authors' primary contributions are: a "system 1" module that consists of a "task-predictor" U-Net for solution proposals and a "distribution-discriminator" meta-learning ResNet that assesses the likelihood of the proposed solutions; a "system 2" module that consists of two adversarial ("auto-competing") models whose iterative refinements to the proposed solution(s) are scored by the "distribution-discriminator". The authors report substantial gains in low-data variants of digit classification and expert medical segmentation tasks – with key results showing comparable performance with end-to-end deep learning systems on fewer than a dozen data samples. Ablation experiments show meaningful contributions from all components in the authors' proposed system.

Overall, I consider the empirical work of this paper quite compelling, with well-validated results that represent an exciting methodological advancement in machine vision of broad and immediate interest to multiple sub-fields and downstream applications. I am somewhat less compelled by what I consider to be the paper's overly general framing, which I think detracts from the otherwise solid set of results precisely by prompting a distracting amount of consideration for the other results that would be necessary to more fully merit the framing. This problem of framing aside, I will say in advance that I would recommend this paper for publication almost as-is and applaud the others accordingly.

Further feedback and comments (primarily intended to further strengthen this already-strong manuscript) below.

Scope of the Claims / Terminology:

"Visual reasoning", as the authors note, is a term that covers a broad array of tasks grounded in visual sensory data. The empirical results of this work, however, apply mainly to sparse-data regimes within two tasks: segmentation and classification. Even excluding visual question-answering (which the authors do by high-lighting the "non-verbal" nature of the visual reasoning that machine vision systems continue to struggle with), there are still a wide variety of visual reasoning tasks this work does not address: keypoint and pose estimation, localization (e.g. odometry), 2D and 3D geometry, path tracking, et cetera. With this in mind, I'd say the results speak more to improvements in dense-prediction (or even just in foundational "perception") more than reasoning, per se.

This limitation, in turn, brings us to the core framing issue: The metaphor of "System 1" and "System 2". I appreciate the reference, and understand where the authors are coming from, but the use of what are (at their core) iterative refinement (denoising) techniques (of which multi-agent / adversarial setups seem also a sub-case) simply does not, in my mind, justify the use of these terms – which entail a massive amount of affordances and associations in a very extensive and diverse array of literatures. I imagine the authors don't particularly wish to engage in the kinds of definitional debates that reference to these terms so often generates, though if they do, there is much more work to be done (and many more results I personally would need to see) to justify the consideration of refinement to be a general case of "System 2"-style visual cognition. Some questions worth considering that I hope will better illustrate my point here:
- Is visual search (which often involves longer time-scales, and deliberative processing) a case of System-2 visual reasoning?
- Are generative adversarial networks (structurally similar in many ways to the self-play reinforcement learning setup the authors propose here) an example of a System 2 "deliberative. slow-thinking" model?
- Is auto-regression a case of "System 2" reasoning since it involves context-specific integration of time-dynamic data?
- Is shape-based visual perception a case of "System 2"? (Many machine learning methods designed to ameliorate the supposed "texture bias" of image classification systems in favor of shape-based recognition involve e.g. recurrence as a solution to sub-problems like border ownership and boundary path-finding.)

Indeed, these examples are a tad pedantic, and I don't necessarily fault the authors for trying to make their results more accessible to a wider audience. The vague metaphorizing of new machine learning methodologies to cognitive psychology + neuroscience is certainly in vogue (see e.g. the recent work on the "Hierarchical Reasoning Model"), but is a bandwagon I

think better to be avoided.

My main recommendation: Change the title to be more about the benefits of iterative / multi-agent / self-refinement in machine vision, drop or dramatically diminish the terminology of “system 1” and “system 2”, and re-focus the introduction on the more directly relevant literatures showcasing issues of visual task-solving in low data regimes. It is here the author’s work most brightly shines!

Suggested Experiments to Justify the Scope:

If the authors do wish to keep the more general scoping of their framework, I think at least a few further experiments are in order, for example:
- Application to spatial reasoning tasks (e.g. on the CLEVR dataset)
- Application to scene understanding (e.g. depth estimation, panoptic segmentation)
... the latter of which, especially, would extend the framework beyond the pixel masks that currently define the experiments, and which could (more detractively) be considered cases of denoising rather than reasoning, per se.

Robustness Checks / Sensitivity Analysis:

In terms of robustness (even if the authors opt for less general framing), perhaps further sensitivity analysis of the refinement operator’s hyperparameters (e.g. pixel-flip proportion) are potentially a key revision necessary to better support the work’s central claims. Cross-validation across different data splits is another route.

Clarifications of Trade-offs and Component Attribution:

To better situate / clarify some of the gains in the empirical work, further details on compute trade-offs and the roles of the pipeline’s different components could be helpful.
While Figure 5 does already address this to a reasonable extent, more detailed scaling curves (compute versus gain-per-sample) and carbon footprint estimations could further emphasize / help the reader better understand the efficiency gains of the proposed method.

Again returning to narrative, what the authors often refer to as “reasoning” is never fully dissociated from known iterative refinement dynamics in other systems. The authors would do well to better address (either with further experiments or in discussion) how their system (conceptually or methodologically) differs from previous / concurrent work such as:

- CRF-RNN (Zheng et al., 2015), which improves pixel-wise labeling in a refinement loop guided by energy minimization
- Diffusion models (e.g. Ho et al, 2020) which perform better with more iterative steps
- Relevant feature selection from pretrained features (e.g. Dvornik et al., 2020)
- HRM / TRM (Wang et al, 2025 / Jolicoeur-Martineau, 2025): Currently popular “reasoning” models showing strong case gains simpler from longer test-time iteration

TLDR, perhaps redundant at this point: The cognitive metaphor of System 1 and System 2 is potentially obscuring the more direct association of this work with these other methods.

Algorithmic Reproducibility of the Key Results:

The repo currently provided by the authors (https://github.com/s-sd/system-II-vision) is insufficient to fully reproduce the key results of the paper, showing only the basic setup of the MNIST digit classification experiments with a basic U-Net and AlexNet model. (Pending deeper inspection, it is unclear to me at the moment if the U-Net in the GitHub repo is comparable to the U-Net described in the methods). Links are provided for the medical datasets, but the repo currently does not provide the data loaders, training scripts, hyperparameter configs, and other sub-modules that would be necessary to fully validate the authors’ setup. (I understand this is a public repo, and that the authors might be waiting for publication to release more code – so this is largely a note I leave at the editor’s request.)

Conclusions + Final Thoughts

Despite the critical feedback above, my overall impression of this work remains largely positive: In short, I think it’s pretty darn solid! I do hope the authors take heed in particular of my notes about framing, but otherwise, I hope even more to see this work published in due course. I congratulate the authors for their progress on this exciting and substantive frontier.

(Remarks on code availability)
(See my comments on "Algorithmic Reproducibility of Key Results" above.)

Version 1:

Reviewer comments:

Reviewer #1

(Remarks to the Author)
Thank the authors for the clarification. It addressed several of my concerns; however, I still have a few remaining comments.

- The paper indicates that the refinement process is a competitive game between two agents with self-play. However, the optimization objective, which is to maximize the segmentation quality score, is optimization-based rather than adversarial-based. The authors should provide theoretical justification for why a competitive formulation will achieve better performance compared to multi-agent reinforcement learning or standard optimization.
- In the System II module, the ground truth for samples is generated based on the discriminator's score. Will any bias in the discriminator lead to model collapse over time?
- The refinement action is based on the XOR flipping. This strategy forces the reasoning process to solely operate on hard binary masks, which may lose valuable uncertainty information. Probability-based approaches may be more reasonable for medical image segmentation rather than the proposed the hard-decision logic.
- Will the proposed method handle multi-object segmentation tasks? For instance, when a refinement improves on lesion but degrades another in a multi-lesion image, what will the proposed System II do?
- The proposed System II is strongly similar to the Test-time Adaptation approaches. Authors should clarify the theoretical difference between these two frameworks.
- From my perspective, the refinement process in System II initializes from the System I's prediction. In extreme cases where the prediction of System I is completely misaligned from the true label, will the XOR refinement operation recover this low-quality first-stage result?
- Authors do not discuss failure cases, such as cases where System II degrades the initial System I prediction. Such analysis will help us build a more comprehensive understanding of the proposed method.

(Remarks on code availability)
Authors have currently provided a working demo for MNIST digit classification (Fig 3 in the revised paper). A README file with sufficient instruction for installing and reproducing this result is provided. I am able to successfully run the provided scripts and I believe the code will be useful for the research community.

Reviewer #2

(Remarks to the Author)
I applaud and thank the authors for their in-depth, comprehensive responses to my feedback / that of other reviews -- and I am pleased to report that on nearly all fronts, I am amply satisfied with the revisions! The sensitivity sweeps in particular very much deepen my confidence in the robustness of the standout results. While I would still take some issue with the System-1 / System-2 framing, I also recognize this is a popularly-used metaphor in the AI/ML literature and don't think that particular gripe clouds the many other excellent contributions of this work, nor overshadows the better scoping the authors provide in this revision. And so, taken together, I recommend accept as-is! Congratulations to the authors on a job well done.

(Remarks on code availability)
The authors have adequately addressed the points raised on reproducibility in my primary review. And so, while I was again not able to reproduce the manuscript results from the provided GitHub repo, I accept the authors' declaration of intent to provide more comprehensively reproducible updates on publication.

Version 2:

Reviewer comments:

Reviewer #1

(Remarks to the Author)
Thank you for the clarification, which addressed several of my earlier concerns. A few minor points remain.

- The +1/−1 relative reward scheme may mitigate absolute drift, but it risks creating a severe information bottleneck. By discretizing the reward signal, the framework loses gradient resolution — a policy update that dramatically improves the mask is treated identically to one yielding marginal gains. This could lead to substantial inefficiency in training. It is unclear whether the current framework has any mechanism to recover from this.
- The refinement procedure bears a close resemblance to iterative denoising in conditional diffusion models. The authors should provide theoretical or empirical justification for why the added complexity of a GAN+RL framework offers a meaningful advantage over a simpler test-time conditional diffusion process initialized on System I's output.
- The choice of N=2 agents warrants explicit discussion. The authors should clarify whether this is an architectural constraint or an empirically motivated decision, and address whether scaling to a larger agent pool degrades performance.
- The robustness of the System I/II adaptation to sample quality remains unverified. Reporting performance standard deviation across multiple sampling seeds would help distinguish genuine improvement from favorable few-shot selection.
- I would like to kindly note that the public repository has not been updated since the last round of review. I would strongly encourage the authors to release the full codebase upon acceptance, enabling the community to reproduce all reported experiments — beyond the existing MNIST demo.

(Remarks on code availability)

I would like to kindly note that the public repository has not been updated since the last round of review. I would strongly encourage the authors to release the full codebase upon acceptance, enabling the community to reproduce all reported experiments — beyond the existing MNIST demo.

Open Access This Peer Review File is licensed under a Creative Commons Attribution 4.0 International License, which permits use, sharing, adaptation, distribution and reproduction in any medium or format, as long as you give appropriate credit to the original author(s) and the source, provide a link to the Creative Commons license, and indicate if changes were made.
In cases where reviewers are anonymous, credit should be given to 'Anonymous Referee' and the source.
The images or other third party material in this Peer Review File are included in the article's Creative Commons license, unless indicated otherwise in a credit line to the material. If material is not included in the article's Creative Commons license and your intended use is not permitted by statutory regulation or exceeds the permitted use, you will need to obtain permission directly from the copyright holder.
To view a copy of this license, visit https://creativecommons.org/licenses/by/4.0/

Reasoning in machine vision: learning to think fast and slow
Point-by-point response to reviews

We thank the reviewers and the editor for their insightful and constructive feedback. We address all comments in a point-by-point manner below, outlining the ‘comment’, ‘response’ and/or ‘action’. Comments from Reviewer 1 are addressed on pages 1-9 and from Reviewer 2 on pages 10-15. Please note that reference numbers used here are separate from the manuscript.

Reviewer 1

Comment: The System II of the method proposed in this paper relies on the probability output of the distribution-discriminator as the sole reward ... As the mask is constantly modified during the reasoning process, the discriminator may be disconnected from the true segmentation quality. This paper does not provide a mechanism to prevent such deviations or drifts.

Action: We agree that it may be possible for the discriminator to be disconnected from the true segmentation quality. Our framework incorporates two mechanisms that maintain alignment between the discriminator and the true task objective. We have now added a brief discussion to the ‘Methodological description’ section of the supplementary materials to discuss both mechanisms, under the paragraph heading ‘Preventing disconnects between the discriminator scores and actual task performance’:

Two mechanisms ensure alignment between the discriminator and the true task objective to be maintained in the reasoning process. First, before System II reasoning begins, both the task-predictor and distribution-discriminator are adapted using a small set of human-labelled samples specific to the target domain. This ensures that predictions from both networks are task-relevant, minimising the difference in objectives between the domain-of-interest and cross-task pre-training. Furthermore, use of the meta-learning algorithm, Reptile, during pre-training, equips the System I module with the ability to become task-relevant with minimal labelled data. Once System II reasoning begins, both networks are held fixed, preventing further drift during the refinement process itself. Second, the reward is defined as the relative improvement in discriminator score between competing refinements, rather than relying on the discriminator’s absolute output. Relative rewards are considerably less sensitive to calibration errors in the discriminator, because what matters is the direction of improvement, not the specific numerical value, which is a common strategy used in RL. This comparison-based formulation therefore mitigates the impact of any drift or noise in the discriminator’s absolute scores. Ablation studies removing the relative reward (auto-competition) are presented in supplementary Tab. 5, which shows that the relative reward formulation offers improved performance. Note that we intentionally used a few samples for adaptation (e.g., 8 samples as shown in Fig. 5c and supplementary Tab. 6). Using a larger adaptation set may further reduce the likelihood of any potential deviation between the discriminator and true segmentation quality. However, our aim was to evaluate the system under a highly sample-efficient regime and demonstrate that strong performance can be achieved even with minimal adaptation data.

Additionally, we have now added an experiment investigating the alignment or correlation between Dice score and the discriminator score when using different amounts of human labelled data for adaptation. This has been added to the 'Ablation and robustness studies' section in the supplementary materials:

To analyse the alignment between task performance and the discriminator score, we investigate the correlation between Dice score and the discriminator score, for the task of prostate cancer segmentation, when using different amounts of human labelled data for adaptation of the System I module. The results are presented in Tab. 6 below, where we measure the Pearson's correlation coefficient (and standard error) between the Dice score (measured between task-predictor labels, from a task-predictor adapted with 32 samples, and human labels) and discriminator score, across 100 human-labelled samples in the holdout set. We observe that approximately 8 samples allow for correlations approaching the level of alignment observed for much larger sets of labelled data.

Human labels	Correlation
0	0.21 ± 0.09
4	0.53 ± 0.09
8	0.81 ± 0.06
12	0.83 ± 0.06
16	0.79 ± 0.06
20	0.84 ± 0.05
24	0.83 ± 0.06
28	0.81 ± 0.06
32	0.80 ± 0.06

Table 1: Impact of human-labelled data for adaptation of the System I module on the alignment between the distribution discriminator scores and actual performance (Dice).

Comment: interested in the adaptation of the method proposed in this paper in classification ... provide more detailed experimental information and discussions

Action: We have now substantially expanded the classification part in the 'Adapting the method for classification or regression' section in the supplementary materials (highlighted in the annotated version of the manuscript):

1. Adding details on the mix-up procedure to generate continuous values for labels.
2. Providing further details and an additional example on the binarisation of multi-class classification problems.
3. Adding a table with detailed comparisons to commonly used methods such as CNNs, trained with the same data as the System II approach, and with the much larger full dataset, showing improved performance with our System II approach.
4. Showing performance improvements compared to other reasoning models such as ChatGPT and Gemini, for a new task of IQ test puzzle correct answer classification, representing a diverse real-world vision task (with experimental details added to the relevant subsection).

Comment: Why does System I use the model of the distribution-discriminator architecture? This architecture is particularly poor at handling small sample situations, and the poor fine-tuning results of small samples might be caused by this reason. Would the effect be much better if it were replaced with a general neural network for segmentation?

Response:

The System I module adopts an adversarial architecture because the two functions it comprises, i.e., the task predictor (which generates solutions) and the distribution discriminator (which assesses the plausibility of that solution) are inherently interdependent. The discriminator must continuously adapt to the evolving predictions of the task predictor, while the task predictor must learn to generate outputs that satisfy the discriminator's learned representation of solution plausibility. This joint optimisation cannot be reproduced by a standalone segmentation network, as it lacks the mechanism to model this mutual dependency.

While adversarial approaches can, in principle, be sensitive to small training sets, in our case the discriminator is not trained to model visual appearance from scratch. Instead, it is pre-trained on other segmentation tasks using the Reptile meta-learning algorithm to allow adaptability to new tasks. Empirically, we did not observe instabilities attributable to small-sample effects in this module.

To test whether a standard segmentation network would perform better in small-sample settings, we reported ablations where adversarial training was removed and the task predictor was trained as a conventional segmentation network. These experiments were reported in supplementary Tab. 5 and detailed in the corresponding supplementary 'Ablation and robustness studies' section. We observed a substantial degradation in performance when adversarial coupling was removed, despite using identical data, hyper-parameters and compute budgets. These findings suggest that the adversarial formulation is not the source of poor fine-tuning performance in small-sample cases. Instead, it is the coupling between prediction and distribution discrimination that provides meaningful supervisory signal beyond what a conventional segmentation network can offer.

In summary, the architecture is adopted because it captures the intrinsic dependency between solution generation and distribution evaluation. Our ablations further confirm that replacing this module with a standard segmentation network leads to inferior performance, even under limited data.

Action: We acknowledge that the above discussion may be of interest to readers and have now added a discussion to the 'Ablation and robustness studies' section in the supplementary materials:

In particular, it is interesting to note that the removal of adversarial training led to a marked reduction in performance, which highlights the interdependent nature of the solution prediction and distribution discrimination. During adversarial training, the discriminator continuously adapts to the evolving predictor outputs, while the predictor updates to better match the learned solution distribution. This coupling enables System I to capture the interdependence between solution quality and distributional plausibility, which is lost when the components are trained separately as conventional neural networks.

Comment: refinement in the method proposed in this paper is calculated through XOR point flipping ... this contradicts the anatomical continuity of actual medical organs or lesions and is prone to generating noisy shapes ... paper does not compare the refinement

based on shape priors, graph optimization or variational methods

Response: We appreciate the reviewer's observation and address the concern in three clarifying points:

1. **Pixel-level XOR refinement may trade explicit continuity priors for flexibility in local regions**
   The XOR mechanism is applied at the pixel level, resulting in pixel-level resolution without enforcing any continuity or smoothness constraints, which may bias the predictions. This design choice is intentional: in cancer segmentation, for example, lesions are often heterogeneous, irregular, fragmented, or multi-focal. Strong shape priors, graph constraints or variational regularisers would enforce smoothness and spatial coherence that may not hold for such pathology, and would risk systematically smoothing out subtle or discontinuous disease patterns. Pixel-level refinement avoids such assumptions and allows the system to represent challenging, less-smooth anatomical or pathological boundaries when the image evidence supports them.

2. **When smoothness is beneficial, it can still be learned implicitly from data rather than enforced a priori.**
   Although we do not impose hand-crafted shape priors, our framework is not unconstrained. During pre-training, the discriminator learns shape statistics across diverse segmentation problems and penalises implausible refinements. In this way, continuity and smoothness (when they are characteristic of the underlying anatomy or pathology) are encouraged implicitly through data, rather than via hard-coded assumptions. This avoids biasing the model toward incorrect anatomical regularity in heterogeneous pathology.

3. **Empirically, continuity is not the dominant source of segmentation error in our setting**
   In practice, the largest performance bottlenecks arise not from local continuity violations but from broader issues such as ambiguous boundaries, artefacts or limited supervision. Our quantitative and qualitative evaluations demonstrate that XOR-based refinements do not produce noisy or anatomically implausible shapes, and that continuity is well preserved in typical cases (see Fig. 4). While we acknowledge that tasks with highly smooth, low-variability anatomy may benefit from explicit shape priors, this is application-dependent. As such, while exploring this direction is interesting for future work, our focus is on performance improvements offered by inference-time optimisation without manual assumptions or constraints.

Action: To the supplementary section 'The definition of refinement' we have now added the following discussion:

Our implementation performs refinement at the pixel level using XOR flipping to allow maximum flexibility in the evolving solution space. While this operates without explicit shape priors or graph/variational constraints, anatomical plausibility is implicitly enforced via the learned distribution-discriminator, which penalises unlikely or anatomically implausible refinements. This avoids imposing hard-coded continuity assumptions, which may conflict with the irregular and heterogeneous morphologies often observed in real cancer pathology.

Comment: comparing the segmentation performance, why do the authors choose U-Net instead of the lighter or multi-scale Swin-UNETR/SegNeXt as baseline? It is suggested to add comparisons with the latest achievements such as SAM-Med and MedKLIP-SegSAM-Med and MedKLIP-Seg

Response: Originally we used the U-Net as the baseline due to it being the most commonly used method for pathological segmentation. Additionally, we compared with the previous best-performing methods found in recent literature. Recent work on more general algorithms may improve inductive biases but gains are often task- and data-dependent.

However, we acknowledge that readers may be interested in more recently emerging models. As such, we have now added comparisons to Swin-UNeTr and SegNeXt to Tab. 3 in the supplementary materials.

The original SAM-Med and MedKLIP-Seg may not be directly comparable to our work as they both are reliant on external inputs, during inference or pre-training. SAM-Med requires prompts for inference, and MedKLIP is designed for knowledge-enhanced pre-training using text-based reports. Since neither prompts nor text reports are available for our model and evaluation datasets, the comparison would not be well-suited for this work. However, if the reviewer is interested, we evaluated the efficacy of SAM-Med on the PROMIS prostate cancer dataset in one of our recent works: Karam et al. 2025. Promptable cancer segmentation using minimal expert-curated data. Medical Image Understanding and Analysis (MIUA) 2025.

Action: As highlighted above, we have now added comparisons to Swin-UNeTr and SegNeXt to Tab. 3 in the supplementary materials.

Comment: System II proposed in this paper merely uses the winning refinement ... Why not consider fusing the two refinements?

Response: We thank the reviewer for this interesting question. In our framework, the candidate solutions are treated as competing, where the competition is intentionally structured to encourage winning by proposing the best possible agent-specific refinement. The choice to use the winning refinement rather than fusing them is motivated by both theoretical and practical considerations, as well as empirical observations. We elaborate these as follows.

1. **Theoretical consideration - stability of the reward signal:** The reward is comparative i.e., the solutions are compared and only the refinement with the higher discriminator score receives a fixed reward. This minimises sensitivity to small fluctuations in the absolute discriminator scores. Fusing refinements would require reward assignment based on absolute discriminator values, making learning more sensitive to calibration noise and score inconsistencies. By contrast, selecting the winning refinement provides a stable, binary reward signal (+1/0), which is known to improve convergence behaviour in RL settings.

2. **Practical consideration - incompatibility between competing refinements:** In many iterations, the two competing proposed refinements are mutually contradictory. In dense segmentation tasks, for example, one refinement may add pixels along the boundary while the other removes pixels from the same region. Such inconsistencies make fusion unreliable, producing inconsistent shapes with conflicting topologies. By selecting only the winning refinement, these incompatibility issues are avoided.

3. **Empirical observation - fusion underperformed in pilot experiments:** In preliminary experiments, we evaluated fusion-based combinations (e.g., union, intersection, soft voting). These approaches did not yield performance gains and frequently degraded segmentation quality, particularly by smoothing out fine lesion boundaries. In contrast, the competitive selection mechanism consistently produced sharper and more anatomically plausible refinements.

4. **Interpretability advantages:** We acknowledge that interpretability can be subjective. With that said, tracking the winning refinement at each iteration results in a clear, stepwise refinement path. This trajectory highlights which regions were repeatedly favoured or rejected, providing an interpretable explanation of the decision process, which was illustrated in our qualitative analysis. Fusing refinements would obscure this interpretability by collapsing multiple hypotheses into a single aggregated update.

Action: To the supplementary materials 'Methodological description' subsection 'The System II module', we have now added a brief statement on the reason for using the winning refinement:

For each refinement step, System II selects the single best hypothesis rather than merging the two proposals. This choice is motivated by firstly the fact that many competing refinements are mutually contradictory (e.g., expanding versus contracting a boundary), where merging would produce semantically inconsistent shapes, and secondly the use of relative, rather than absolute, discriminator-based reward assignment, which provides stability against small fluctuations in absolute discriminator scores.

Comment: hyperparameters such as p and i_{end} are obtained through empirical grid search ... Why not conduct adaptive design according to the task ... It is suggested that the analysis data of hyperparameters be provided

Action: In our experiments, we noticed that the optimal values for the hyper-parameters e.g., p and i_{end}, did not vary across our evaluation datasets (see added analysis below). We note that this may be because of a strong dependence of these on the image size. Fully-data-driven adaptive design for the task may risk introducing additional instability, where for fixed-resolution datasets considered in this work, the set hyper-parameters proved consistently optimal across tasks. However, we fully agree that adaptive hyper-parameter design could be beneficial in tasks with variable image resolution, markedly different ROI sizes, or clinical workflows requiring dynamic computation budgets. We have now added the analysis of hyper-parameters to Tab. 8 in the supplementary materials, accompanied by a brief discussion on: 1) dependence of optimal values on the image size and 2) the utility of task-specific hyper-parameter settings, in cases where images or ROI sizes are different across tasks:

The analysis of hyper-parameters p (the proportion of pixels to flip per iteration) and i_{end} is presented in Tab. 8. Note that we observed a high dependence of the optimal values for both hyper-parameters on the input size or number of pixels in the image P. As such, we defined i_{end} as a function of P. As the input size stays consistent across datasets in our work, we observed a consistent optimal value for both p and i_{end} across the evaluation datasets. However, for other datasets, in future work, these parameters may need to be tuned according to the input or ROI size.

Proportion p	Termination i_{end}	Prostate	Liver	Pancreas
0.001	$p \times P \times 1$	56.8±5.7	74.3±8.8	-
0.005	$p \times P \times 1$	60.4±4.9	79.1±9.2	62.9±8.8
0.010	$p \times P \times 1$	61.7±5.8	81.5±9.7	65.2±9.2
0.050	$p \times P \times 1$	61.3±6.3	78.7±9.1	62.4±9.9
0.100	$p \times P \times 1$	50.4±7.8	65.3±11.7	-
0.010	$p \times P \times 1$	61.7±5.8	81.5±9.7	-
0.010	$p \times P \times 10$	60.6±5.4	83.7±9.5	65.6±9.1
0.010	$p \times P \times 100$	63.0±4.8	85.3±8.6	66.2±9.4
0.010	$p \times P \times 1000$	58.4±5.9	80.5±9.5	61.1±8.7
0.010	$p \times P \times 10000$	54.1±6.7	78.4±9.4	-

Table 2: Impact of hyper-parameters on performance for models adapted using 8 labelled samples.

Comment: provide the computational costs of Liver, Pancreas, Colon, Kidney and other tasks involved in this paper ... suggested to give a comparison of the reasoning delay time of different methods

Action: We have now added these, along with the reasoning times (given the specified number of adaptation samples / experience), to the section 'Computational cost' in the supplementary materials.

Task	GPU Hours								Power (W)	Carbon (kg CO2 / GPU hour)
	Pre-Train	Adapt	Inference / Reasoning Time							
			Experience (Number of Samples)							
			0	8	16	24	32	40		
Prostate	1152	16	41	36	24	16	6	6	300 W	0.13
Liver	1152	18	37	34	20	8	8	7	300 W	0.13
Pancreas	1152	13	42	39	19	9	7	8	300 W	0.13
Colon	1152	15	33	30	11	3	4	3	300 W	0.13
Kidney	1152	21	29	23	17	10	4	2	300 W	0.13

Table 3: Carbon footprints estimated on Nvidia V100 GPUs.

Comment: appropriately add one or two evaluation metrics, such as mIoU

Action: We have now added a new Tab. 4 to the supplementary materials with mIoU reported:

Method	Pre-Train	Labels	Prostate	Liver	Pancreas	Colon	Kidney
System II	Yes	8	45.3±5.4	**74.4±13.1**	43.8±10.0	**48.3±11.8**	**60.8±8.1**
System II	Yes	12	**46.0±5.1**	72.3±15.1	46.6±9.6	46.3±10.9	59.9±7.5
System II	Yes	16	45.8±6.2	72.9±13.6	**49.5±10.5**	47.8±12.0	58.9±7.8
UNet [20]	Yes	8	14.2±3.6	19.5±7.2	14.0±5.5	21.1±7.8	18.8±4.4
UNet [20]	Yes	12	16.6±3.6	25.2±8.9	18.5±6.5	19.3±8.3	20.6±3.9
UNet [20]	Yes	16	17.5±3.2	27.5±7.6	29.0±7.2	26.3±8.9	21.4±4.2
UNet [20]	Yes	>100	26.3±4.7	59.0±12.3	39.0±8.8	36.9±11.2	56.0±8.3
SUNeTR [9]	Yes	8	14.7±3.5	21.8±7.9	15.1±5.8	21.1±7.2	20.6±4.8
SUNeTR [9]	Yes	12	16.8±4.0	24.5±8.1	20.2±7.1	22.2±8.7	21.3±4.8
SUNeTR [9]	Yes	16	20.8±3.6	29.8±8.3	30.0±7.7	29.3±8.9	27.7±4.7
SUNeTR [9]	Yes	>100	26.5±4.3	62.2±13.4	40.2±8.7	35.9±10.3	54.8±7.1
SegNeXt [8]	Yes	8	13.3±2.9	18.3±8.3	14.9±6.5	19.4±8.5	18.5±4.7
SegNeXt [8]	Yes	12	17.4±3.6	26.1±7.7	19.6±6.0	23.7±8.6	21.3±4.6
SegNeXt [8]	Yes	16	20.0±4.2	28.8±8.5	31.9±8.1	27.3±9.2	31.4±5.3
SegNeXt [8]	Yes	>100	25.2±3.8	58.7±13.2	41.0±8.9	39.6±10.4	54.9±7.0
UNet [20]	No	8	10.1±3.5	11.6±5.5	11.4±6.0	16.5±8.0	18.2±4.6
UNet [20]	No	12	12.8±2.9	15.9±6.5	20.4±6.7	17.7±8.0	19.3±3.7
UNet [20]	No	16	15.5±3.3	21.0±7.7	26.1±6.8	25.7±8.1	24.5±4.9
UNet [20]	No	>100	23.2±3.7	59.4±13.2	37.0±9.3	38.1±9.7	54.1±6.6
Dv2 [17]	Yes	8	23.8±3.8	23.0±6.9	22.7±6.5	22.0±7.7	23.5±4.7
Dv2 [17]	Yes	12	23.4±3.3	24.5±6.7	22.9±6.7	22.7±7.9	23.3±3.9
Dv2 [17]	Yes	16	23.8±4.3	26.3±7.7	24.0±7.1	24.4±8.9	25.7±4.5
Dv2 [17]	Yes	>100	25.6±4.4	58.1±12.9	38.8±8.8	36.4±9.6	56.9±8.4
USeg [3]	Yes	8	16.0±3.6	-	19.9±5.9	24.3±9.2	-
USeg [3]	Yes	12	21.0±4.2	-	21.0±6.5	25.4±8.9	-
USeg [3]	Yes	16	22.0±3.5	-	25.5±6.9	27.3±8.8	-
USeg [3]	Yes	>100	26.7±4.1	-	43.6±9.8	44.4±10.5	-
nnUNet [12]	No	>100	26.8±4.5	-	-	-	-
CLIP [14]	Yes	>100	-	65.8±11.1	45.2±10.3	46.1±11.3	-
ASeg [16]	No	>100	-	-	-	-	**61.8±7.2**

Table 4: The mean intersection over union (mIoU) metric reported for cancer lesion segmentation, corresponding to the above Tab. 3 (where Dice was reported).

Comment: information in the main text and the appendix is scattered

Response: The journal guidelines state that Methods and Results in the main manuscript should be succinct (less than 3000 words), as such, in our main text we provided high-level descriptions; with mathematical notation and detailed results presented in the supplementary materials. We acknowledge that this may be confusing for more technically inclined readers, however, it may allow for broad readability of the main manuscript.

Action: To improve the information flow, we have now re-done the section headings into 'Introduction', 'Methods', 'Results' and 'Conclusion' to match a more conventional layout. We have also added pointers to the supplementary materials, where appropriate, in the main manuscript.

Comment: test the robustness of the proposed method against noisy data or outliers

Action: We have now added the following new experiment to 'Impact of annotation noise' under 'Ablation and robustness studies' in the supplementary materials:

We study the impact of noisy human annotated data on the performance of our proposed method compared to deep learning. The detailed quantitative results are presented in Tab. 7. Note that we added random deformations to the segmentation labels to simulate human error in annotation, the biggest source of noise in our data. The segmentation masks were deformed using a grid of 3×3 control points, each with a random displacement of 0-40 pixels or 0-20mm, with dense displacements computed using linear interpolation. The images themselves were kept unchanged, as they already contain real-world noise sources (e.g., motion artefacts, probe variability and sensor noise), and because realistic simulation of imaging noise remains an open research problem without standardised models. Additionally, For different proportions of noisy adaptation samples, we compared the proposed method with a deep-learning-based segmentation baseline. As shown below, both methods experience performance degradation as noise increases, but our approach shows a more gradual decline, indicating better robustness to annotation outliers.

Percentage corrupted	System II	DL - Pre-trained
0	62.7±5.7	40.6±4.9
25	60.1±5.2	31.4±5.9
50	51.3±5.9	26.8±5.6
75	40.2±5.4	22.6±4.8
100	30.2±5.2	21.3±5.5

Table 5: Studying the impact of noisy data on performance for the prostate cancer segmentation task. The 'percentage corrupted' refers to the percentage of adaptation data to which noise was added out of a total of 32 adaptation samples.

Reviewer 2

Comment: Visual reasoning is a term that covers a broad array of tasks grounded in visual sensory data ... empirical results of this work, however, apply mainly to sparse-data regimes within two tasks: segmentation and classification ... results speak more to improvements in dense-prediction (or even just in foundational "perception") more than reasoning

main recommendations: re-focus the introduction on the more directly relevant literatures showcasing issues of visual task-solving in low data regimes ... better address (either with further experiments or in discussion) how their system (conceptually or methodologically) differs from previous / concurrent work ... CRF-RNN (Zheng et al., 2015) ... Diffusion models (e.g. Ho et al, 2020) ... feature selection from pretrained features (e.g. Dvornik et al., 2020) ... HRM / TRM (Wang et al, 2025 / Jolicoeur-Martineau, 2025)

Response: We thank the reviewer for the thoughtful comment. We agree that visual reasoning is a broad term covering heterogeneous tasks. Our use of the term follows its recent usage in deep learning, where reasoning often denotes the use of additional inference-time computation to refine or improve predictions, rather than a claim to emulate the full breadth of human visual reasoning e.g., [23, 21, 11, 7, 15, 2]. Our empirical contribution is focused on scenarios where such inference-time refinement is most impactful, namely data-scarce dense-prediction and classification tasks, including segmentation of heterogeneous pathology.

Our intention was not to generalise beyond these settings, but to show that iterative inference-time refinement can serve as a principled mechanism for improving performance where conventional deep learning or foundation models are constrained by limited data or fixed inference compute. We appreciate that some parts of the introduction could better reflect this scope, and have revised the manuscript accordingly, as highlighted in the 'Action' below.

Action: Firstly, we have revised the introduction and the abstract to make the following changes (exact changes highlighted in the annotated version of the manuscript):

1. Clearly define machine visual reasoning as inference-time compute used to refine solutions and improve performance, with relevant references to recent literature.

2. Reduce the broad terminology of 'visual reasoning' in favour of 'learning in data-scarce regimes' and 'inference-time compute', to more accurately reflect the scope of the empirical work.

3. Add context explaining that our experiments focus on a few representative sparse-data tasks where performance could be improved through machine visual reasoning.

Secondly, we have added a paragraph in 'Section Progress towards inference-time compute capabilities in machines' comparing our method with the categories of approaches highlighted by the reviewer.

Several existing approaches aim to refine initial visual predictions, marking a step toward inference-time compute. Structured-prediction methods such as CRF-based refinements [25] enhance spatial coherence through hand-crafted constraints and a fixed number of update steps. Diffusion-based iterative approaches [10] also refine predictions but follow predetermined generative trajectories, with fixed steps, and typically rely on large-scale datasets for training.

Other methods improve data efficiency by selecting or adapting pretrained features [4], or by refining internal representations through hierarchical modules [22, 13], though their inference-time computation is fixed once trained. In contrast, inference-time compute analogous to System II thinking would allow performance to improve with added unbounded computation/ thinking time, even when labelled data is scarce.

Finally, in the discussion in section 'Results' we have now clarified the scope of our empirical work:

Our experiments focus on representative data-scarce vision tasks that are clinically and scientifically important, where conventional deep learning often struggles. We do not aim to cover the full breadth of visual reasoning. Instead, we show that inference-time computation, via competitive hypothesis refinement, can reliably improve performance when additional labelled data are limited or unavailable. These results illustrate a broader principle: increasing computation at inference can serve as an effective alternative to increasing training data, with potential to extend to other visual tasks in future work.

Comment: framing issue: The metaphor of "System 1" and "System 2" ... the use of what are (at their core) iterative refinement (denoising) techniques (of which multi-agent / adversarial setups seem also a sub-case) simply does not, in my mind, justify the use of these terms – which entail a massive amount of affordances and associations in a very extensive and diverse array of literatures

questions worth considering that I hope will better illustrate my point ... is visual search ... generative adversarial networks ... auto-regression ... shape-based visual perception ... case of System II? main recommendation ... title to be more about the benefits of iterative / multi-agent / self-refinement in machine vision ... dramatically diminish the terminology of "system 1" and "system 2" ... re-focus the introduction on the more directly relevant literatures showcasing issues of visual task-solving in low data regimes at least a few further experiments are in order ... spatial reasoning ... scene understanding

Response: We thank the reviewer for this thoughtful framing comment. We fully appreciate that the terms System I and System II carry strong associations from cognitive psychology and must be used with precision. Our intention was not to imply a literal cognitive model, nor to suggest that all iterative refinement qualifies as System II thinking. Instead, we used dual-process terminology as a conceptual analogy to describe the separation between fast, feed-forward inference and slower, compute-intensive refinement that can continue improving a solution.

This dual-process framing has been adopted in recent machine learning and deep learning research (e.g., [21, 2, 24, 6, 5, 18, 19]). As such, use of these terminologies allows for broad readership.

That said, we recognise the reviewer's concern that the metaphor may read as broader than our empirical scope. We have therefore narrowed and clarified the language around human cognition, ensuring that it is presented as a high-level analogy and not as a scientific claim with psychological correspondence. We have also refocused several aspects and made clear the relevance to existing literature on visual task-solving in data-scarce settings. The specific changes are highlighted in the 'Action' below.

Regarding whether iterative visual search, GANs, autoregression, or shape-based perception should themselves be called System II, we agree that this highlights the ambiguity of the terminology when applied too broadly. For clarification, our definition for System II in ma-

chines encompasses only methods that use unbounded inference-time compute to improve performance, with inference-time methodologies not fixed or learnt based on extensive datasets. The reviewer's questions further support narrowing our metaphorical framing and adding more rigorous definitions, which we have now done as highlighted in the 'Action'.

Finally, we appreciate the reviewer's suggestion to examine additional tasks such as spatial reasoning or scene understanding, which are indeed exciting directions. Our empirical objective in the main manuscript is more focused , i.e., to examine whether inference-time refinement can reliably improve performance in sparse-data regimes. We have now clarified this in the text and narrowed the general framing. In addition, to demonstrate the generality of the mechanism, we have now included two experiments, as requested by the reviewer, in the supplementary materials as highlighted in the 'Action' below. For demonstrating spatial reasoning, we have added a classification task for an IQ test and, for scene understanding, we have added a regression task focusing on monocular depth estimation. These results are not central to the core claims of the paper, but are provided for interested readers and to illustrate the method's potential beyond dense prediction.

Action: Firstly, we have revised the abstract and introduction (exact changes highlighted in the annotated version of the manuscript):

1. Reduced the System I and System II metaphor, replacing with more specific terminology, such as 'single-forward pass', 'fixed inference' for System I, and 'inference-time compute', 'iterative refinement' for System II.

2. Added concise definitions for analogies for System I and System II in machines, where System I is defined as a single feed forward pass mechanism at inference time and System II is defined as inference-time compute used to improve performance, where inference-time mechanisms are not fixed or learnt based on extensive task-specific data.

3. Clarify that the dual-process terminology is only a high-level analogy, with the method being technically comparable to relevant recent works on iterative refinement in low-data learning paradigms (as also noted in the response to the first comment).

Secondly, in 'Results' we have now clarified explicitly that our work does not claim to model human cognition and the empirical scope is on data-independent performance improvement in data-scarce settings:

We emphasise that the use of dual-process terminology serves only as a high-level conceptual analogy for separating fast feed-forward prediction from slower unbounded iterative refinement, and is not intended as an emulated cognitive model. Our empirical work shows the performance improvements, regardless of the availability of task-specific data, possible by enabling inference-time compute in vision tasks where data are scarce.

As noted above, our experiments focus on a few vision tasks, chosen because they represent clinically and scientifically important settings where labelled data are scarce and conventional deep-learning methods underperform. We do not claim to address the full breadth of visual reasoning, which spans diverse domains and task families. Instead, our results demonstrate that inference-time compute or reasoning, implemented through competitive hypothesis refinement, can reliably improve performance in settings where additional labelled data are difficult or impossible to obtain. We view these studies as exemplars of a broader principle, where extending

computation at inference can serve as a powerful alternative to expanding training datasets, and may generalise to a wider range of visual tasks in future work.

Finally, we have added the following new experiments, focusing on spatial reasoning through IQ test puzzles and scene understanding through depth estimation, in the revised supplementary materials under the section 'Adapting the method for classification or regression'. The results table is presented below and the corresponding experimental descriptions are added to the manuscript directly (highlighted in the annotated version of the manuscript):

Method	MNIST classification	IQ classification	Depth regression
	Accuracy	Accuracy	RMSE
CNN (same data)	0.829	0.244	0.514
MLP (same data)	0.746	0.239	0.535
CNN (full data)	0.931	0.271	0.379
MLP (full data)	0.927	0.261	0.443
ChatGPT5 (4-shot prompt)	-	0.264	-
Gemini2.0plus (4-shot prompt)	-	0.278	-
PixelFormer [1] (same data)	-	-	0.412
PixelFormer [1] (full data)	-	-	0.322
Ours	0.953	0.512	0.322

Table 6: Results for classification and regression tasks using the proposed framework. 'Same data' refers to compared methods that use the same training and adaptation data as the proposed method, whereas 'full data' refers to the use of the entire dataset for training for the compared methods.

Comment: further sensitivity analysis of the refinement operator's hyperparameters (e.g. pixel-flip proportion) ... cross-validation across different data splits

Response: We have now added the sensitivity analysis of the pixel-flip proportion p and the number of steps until termination i_{end} to 'Ablation and robustness studies' in the supplementary materials, please see 'Action' below for further details.

Regarding cross-validation, conducting full k-fold evaluation would require repeating both training and inference-time refinement for each split, which is computationally expensive given that our method involves substantial inference-time compute. Cross-validation is therefore more well-suited to to smaller data regimes or methods where training and inference require lesser compute. Instead, we follow established practice in recent literature on these datasets, by reporting results on dedicated hold-out sets of comparable size. We also report performance variance across the test set, providing a statistically-powered, meaningful estimate of robustness without the prohibitive cost of full cross-validation.

Action: We have added the analysis of hyper-parameters to 'Ablation and robustness studies' in the supplementary materials:

The analysis of hyper-parameters p (the proportion of pixels to flip per iteration) and i_{end} is presented in Tab. 8. Note that we observed a high dependence of the optimal values for both hyper-parameters on the input size or number of pixels in the image P. As such, we defined i_{end} as a function of P. As the input size stays consistent across datasets in our work, we observed a

consistent optimal value for both p and i_{end} across the evaluation datasets. However, for other datasets, in future work, these parameters may need to be tuned according to the input or ROI size.

Proportion p	Termination i_{end}	Prostate	Liver	Pancreas
0.001	$p \times P \times 1$	56.8±5.7	74.3±8.8	-
0.005	$p \times P \times 1$	60.4±4.9	79.1±9.2	62.9±8.8
0.010	$p \times P \times 1$	61.7±5.8	81.5±9.7	65.2±9.2
0.050	$p \times P \times 1$	61.3±6.3	78.7±9.1	62.4±9.9
0.100	$p \times P \times 1$	50.4±7.8	65.3±11.7	-
0.010	$p \times P \times 1$	61.7±5.8	81.5±9.7	-
0.010	$p \times P \times 10$	60.6±5.4	83.7±9.5	65.6±9.1
0.010	$p \times P \times 100$	63.0±4.8	85.3±8.6	66.2±9.4
0.010	$p \times P \times 1000$	58.4±5.9	80.5±9.5	61.1±8.7
0.010	$p \times P \times 10000$	54.1±6.7	78.4±9.4	-

Table 7: Impact of hyper-parameters on performance for models adapted using 8 labelled samples.

Comment: further details on compute trade-offs and the roles of the pipeline's different components could be helpful ... more detailed scaling curves (compute versus gain-per-sample) and carbon footprint estimations

Action: The average performance across the dataset and its variation with compute time, number of adaptation samples and number of solved samples were presented in Fig. 5c. We have now also computed the detailed scaling curves, as requested, which have been added to 'Impact of compute on per-sample performance' in the supplementary materials:

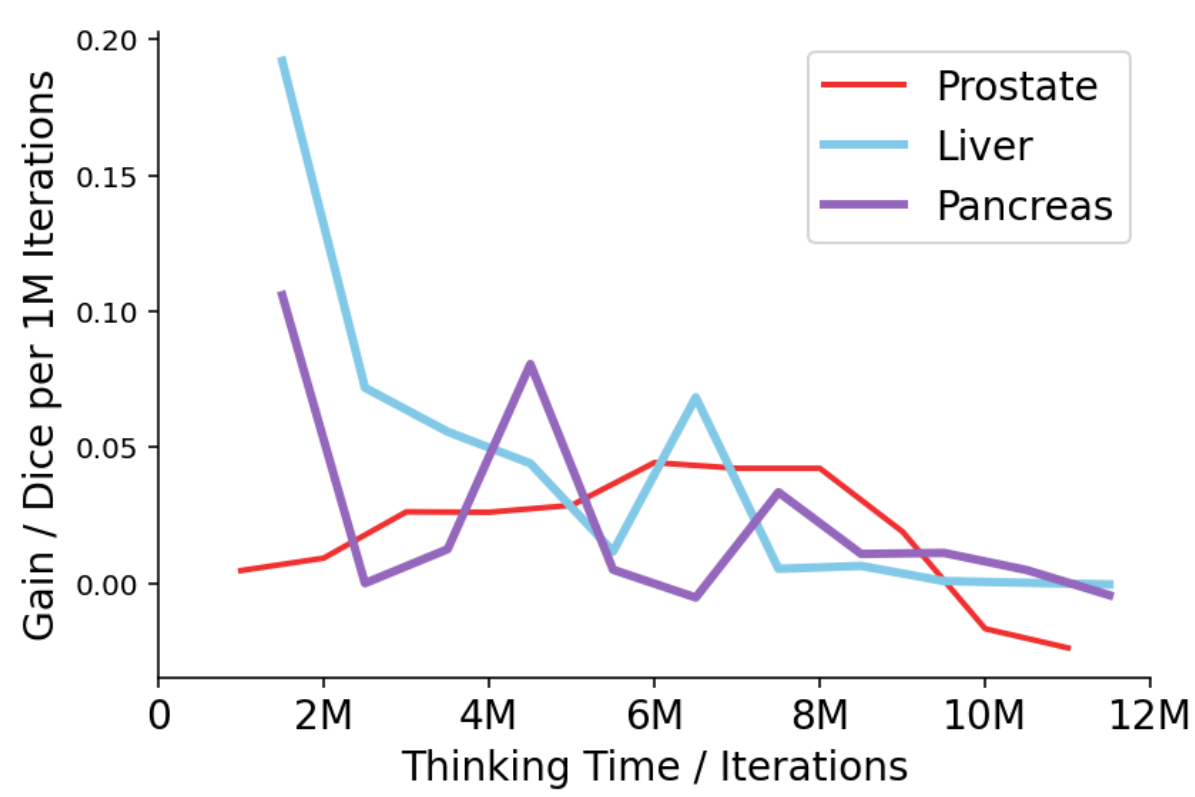

Figure 1: Performance gain per-sample against number of compute iterations

Fig. 7 shows the performance gain per-sample against the number of iterations. We observe an overall reduction in gain, towards zero, with an increasing number of iterations. This highlights that the proposed auto-competing mechanism converges to stable values as the refinement in the System II module progresses.

The carbon footprints and times taken for training and inference/ reasoning, for all tasks, are now added to 'Computational cost' in the supplementary materials:

Task	GPU Hours								Power (W)	Carbon (kg CO2 / GPU hour)
	Pre-Train	Adapt	Per-Sample Inference							
			Experience (Number of Samples)							
			0	8	16	24	32	40		
Prostate	1152	16	41	36	24	16	6	6	300 W	0.13
Liver	1152	18	37	34	20	8	8	7	300 W	0.13
Pancreas	1152	13	42	39	19	9	7	8	300 W	0.13
Colon	1152	15	33	30	11	3	4	3	300 W	0.13
Kidney	1152	21	29	23	17	10	4	2	300 W	0.13

Table 8: Carbon footprints estimated on Nvidia V100 GPUs.

Comment: repo currently provided ... showing only the basic setup of the MNIST digit classification experiments with a basic U-Net and AlexNet model ... does not provide the data loaders, training scripts, hyperparameter configs, and other sub-modules that would be necessary to fully validate ... I understand this is a public repo, and that the authors might be waiting for publication to release more code

Response: We appreciate the reviewer's emphasis on reproducibility. The current public repository provides a working example for the MNIST experiment, and we fully agree that expanding the available scripts and configurations will further support validation. We are committed to releasing all components that can be publicly shared, and the repository is intended as a living resource that we will continue to develop, as the reviewer suggests.

We note that some elements of the full pipeline (e.g., processed data) cannot be released verbatim due to licensing constraints associated with the included clinical datasets. In addition, several modules require substantial compute resources at inference and would require significant adaptation by the users. To address this, we will release all components as they are, in addition to some lightweight, reproducible examples and detailed configuration files that mirror the behaviour of the full system while remaining accessible to the community.

Action: Upon acceptance/publication, we will update the repository with the expanded materials, including data loaders, training scripts, hyper-parameter configurations, and additional example experiments. We are committed to maximising transparency and open-science practice within the limits of data-governance constraints.

References

[1] Ashutosh Agarwal and Chetan Arora. "Attention attention everywhere: Monocular depth prediction with skip attention". In: *Proceedings of the IEEE/CVF Winter Conference on Applications of Computer Vision*. 2023, pp. 5861–5870.

[2] Thomas Anthony, Zheng Tian, and David Barber. "Thinking fast and slow with deep learning and tree search". In: *Advances in neural information processing systems* 30 (2017).

[3] Victor Ion Butoi et al. "Universeg: Universal medical image segmentation". In: *Proceedings of the IEEE/CVF International Conference on Computer Vision*. 2023, pp. 21438–21451.

[4] Nikita Dvornik, Cordelia Schmid, and Julien Mairal. "Selecting relevant features from a multi-domain representation for few-shot classification". In: *European conference on computer vision*. Springer. 2020, pp. 769–786.

[5] Francesco Fabiano et al. "Fast and slow planning". In: *arXiv preprint arXiv:2303.04283* (2023).

[6] Marianna Bergamaschi Ganapini et al. "Combining Fast and Slow Thinking for Human-like and Efficient Decisions in Constrained Environments." In: *NeSy*. 2022, pp. 171–185.

[7] Daya Guo et al. "DeepSeek-R1: Incentivizing Reasoning Capability in LLMs via Reinforcement Learning". In: *arXiv preprint arXiv:2501.12948* (2025).

[8] Meng-Hao Guo et al. "Segnext: Rethinking convolutional attention design for semantic segmentation". In: *Advances in neural information processing systems* 35 (2022), pp. 1140–1156.

[9] Ali Hatamizadeh et al. "Swin unetr: Swin transformers for semantic segmentation of brain tumors in mri images". In: *International MICCAI brainlesion workshop*. Springer. 2021, pp. 272–284.

[10] Jonathan Ho, Ajay Jain, and Pieter Abbeel. "Denoising diffusion probabilistic models". In: *Advances in neural information processing systems* 33 (2020), pp. 6840–6851.

[11] Arian Hosseini et al. "V-star: Training verifiers for self-taught reasoners". In: *arXiv preprint arXiv:2402.06457* (2024).

[12] Fabian Isensee et al. "nnU-Net: a self-configuring method for deep learning-based biomedical image segmentation". In: *Nature methods* 18.2 (2021), pp. 203–211.

[13] Alexia Jolicoeur-Martineau. "Less is more: Recursive reasoning with tiny networks". In: *arXiv preprint arXiv:2510.04871* (2025).

[14] Jie Liu et al. "Clip-driven universal model for organ segmentation and tumor detection". In: *Proceedings of the IEEE/CVF International Conference on Computer Vision*. 2023, pp. 21152–21164.

[15] Runze Liu et al. "Can 1B LLM Surpass 405B LLM? Rethinking Compute-Optimal Test-Time Scaling". In: *arXiv preprint arXiv:2502.06703* (2025).

[16] Andriy Myronenko et al. "Automated 3D Segmentation of Kidneys and Tumors in MICCAI KiTS 2023 Challenge". In: *International Challenge on Kidney and Kidney Tumor Segmentation*. Springer, 2023, pp. 1–7.

[17] Maxime Oquab et al. “Dinov2: Learning robust visual features without supervision”. In: *arXiv preprint arXiv:2304.07193* (2023).

[18] Quang Pham, Chenghao Liu, and Steven CH Hoi. “Continual learning, fast and slow”. In: *IEEE Transactions on Pattern Analysis and Machine Intelligence* (2023).

[19] Biqing Qi et al. “Interactive continual learning: Fast and slow thinking”. In: *Proceedings of the IEEE/CVF Conference on Computer Vision and Pattern Recognition*. 2024, pp. 12882–12892.

[20] Olaf Ronneberger, Philipp Fischer, and Thomas Brox. “U-net: Convolutional networks for biomedical image segmentation”. In: *Medical image computing and computer-assisted intervention–MICCAI 2015: 18th international conference, Munich, Germany, October 5-9, 2015, proceedings, part III 18*. Springer. 2015, pp. 234–241.

[21] DiJia Su et al. *Dualformer: Controllable Fast and Slow Thinking by Learning with Randomized Reasoning Traces*. 2024. arXiv: `2410.09918 [cs.AI]`. URL: `https://arxiv.org/abs/2410.09918`.

[22] Guan Wang et al. “Hierarchical Reasoning Model”. In: *arXiv preprint arXiv:2506.21734* (2025).

[23] Jason Wei et al. “Chain-of-thought prompting elicits reasoning in large language models”. In: *Advances in neural information processing systems* 35 (2022), pp. 24824–24837.

[24] Suhang You et al. *Biochemical Prostate Cancer Recurrence Prediction: Thinking Fast & Slow*. 2024. arXiv: `2409.02284 [cs.CV]`. URL: `https://arxiv.org/abs/2409.02284`.

[25] Shuai Zheng et al. “Conditional random fields as recurrent neural networks”. In: *Proceedings of the IEEE international conference on computer vision*. 2015, pp. 1529–1537.

Reasoning in machine vision: learning to think fast and slow
Point-by-point response to second round reviews

We thank the reviewers and the editor for their insightful and constructive feedback in this second round of reviews. We address all comments in a point-by-point manner below, outlining the 'comment', 'response' and/or 'action'.

Comments from Reviewer 1 are addressed on pages 1-7 and from Reviewer 2 on page 8.

Reviewer 1

Comment: Thank the authors for the clarification. It addressed several of my concerns; however, I still have a few remaining comments.

Response: We thank the reviewer for their continued engagement with the manuscript and for the constructive feedback. We address the remaining comments point-by-point below.

Comment: The paper indicates that the refinement process is a competitive game between two agents with self-play. However, the optimization objective, which is to maximize the segmentation quality score, is optimization-based rather than adversarial-based. The authors should provide theoretical justification for why a competitive formulation will achieve better performance compared to multi-agent reinforcement learning or standard optimization.

Response: We thank the reviewer for this thoughtful comment. The objective of the System II module is indeed to optimise segmentation quality with respect to the discriminator score, which serves as a learned proxy for the true task metric at inference time. The competitive formulation serves as a structural mechanism for stabilising optimisation under an imperfect reward signal.

Rather than directly maximising the absolute discriminator score, the framework evaluates two alternative refinements and enacts and rewards only the higher-scoring proposal. Compared to direct standard optimisation, this relative reward formulation reduces sensitivity to calibration noise in the absolute discriminator scores, as only the direction of improvement is considered rather than the exact magnitude. The optimisation using this relative reward serves as a stable binary reinforcement signal minimising the impact of any small fluctuations in the absolute discriminator scores, which the standard direct optimisation cannot accomplish.

In principle, this approach could be extended to a broader multi-agent setting with multiple simultaneous competing refinements. However, such formulations would require ranking or weighting multiple discriminator scores, increasing reliance on absolute score calibration and amplifying the impact of noise in the learned discriminator. Restricting the process to pairwise competition, with optimisation only considering direction of improvement rather than magnitude, ensures that the impact of errors is minimised while still encouraging exploration through explicit hypothesis comparison.

We therefore view the competitive structure not as adversarial in a game-theoretic sense, but

as a principled design choice for robust iterative optimisation when the reward function itself is imperfect. Importantly, we do not claim superiority over all optimisation strategies, but rather one of our contributions is a specific practical formulation for inference-time compute/reasoning in vision applications.

We have clarified the distinctions between the optimisation strategies in the revised manuscript, as outlined below.

Action: We have added the following to 'Optimising the competitors during the auto-competing process' paragraph in the 'The System II module - for an iterative competition between solution refinements' section of the supplementary materials:

Although the objective of the System II module is to improve segmentation quality, refinement is structured as pairwise competition rather than direct maximisation of the discriminator score. As the discriminator provides a learned proxy rather than the true task metric at inference, relying on absolute score optimisation can be sensitive to calibration noise. Instead, at each iteration two candidate refinements are evaluated comparatively, and only the higher-scoring proposal is enacted and rewarded. This relative reward formulation produces a stable binary reinforcement signal and reduces sensitivity to small fluctuations in the absolute discriminator outputs. Limiting the process to pairwise competition, rather than multiple simultaneous comparisons, maintains a well-conditioned optimisation signal (less impacted by absolute discriminator scores) by avoiding the need to rank or weight multiple competing refinements.

Comment: In the System II module, the ground truth for samples is generated based on the discriminator's score. Will any bias in the discriminator lead to model collapse over time?

Response: We thank the reviewer for raising this important point. Our framework incorporates two design choices that mitigate the impact of potential bias in the discriminator.

First, before System II reasoning begins, both the task-predictor and distribution-discriminator are adapted to the target domain using a set of human-labelled samples. This anchors the discriminator to the true task objective and ensures that its scores reflect the characteristics of the task of interest. As reported in supplementary Table 6, we observed that approximately 8 adaptation samples lead to strong alignment (measured as Pearson's correlation coefficient > 0.7) between the discriminator score and segmentation Dice score for the prostate cancer segmentation task. This highlights the importance of the adaptation stage in calibrating the learned discriminator to reduce biases before reasoning begins. During the reasoning process itself, the discriminator is held fixed (no model updates), ensuring that no further bias is introduced over time during System II refinement.

Second, the reward used during refinement is defined comparatively rather than absolutely. At each iteration, two competing refinements are evaluated and only the higher-scoring proposal is rewarded and enacted. This relative formulation reduces sensitivity to calibration errors in the discriminator because optimisation depends on the direction of improvement rather than the exact score value. Consequently, small biases in the discriminator are unlikely to accumulate over time during the iterative reasoning process.

Empirically, the correlations between discriminator and Dice scores were strong (Pearson's correlation coefficient >0.78) for all tested tasks when using 8 samples for adaptation. This suggests that the learned discriminator in our experiments remained aligned with the true task objective.

Action: We have now updated the supplementary materials paragraph 'Preventing disconnects between the discriminator scores and actual task performance', to reflect how the two mechanisms mitigate bias accumulation in the iterative reasoning process:

For the adaptation using human-labelled samples from the task-of-interest, we added the following:

This ensures that predictions from both networks are task-relevant, minimising the difference in objectives between the domain-of-interest and cross-task pre-training. Furthermore, use of the meta-learning algorithm, Reptile, during pre-training, equips the System I module with the ability to become task-relevant with minimal labelled data. Once System II reasoning begins, both networks are held fixed, preventing further drift during the refinement process itself. As neither the task-predictor nor the discriminator is updated during System II reasoning, the iterative refinement process cannot introduce or amplify biases in the learned reward signal.

For the relative reward signal, we added the following:

This comparison-based formulation mitigates the impact of calibration errors in the discriminator because optimisation depends on the direction of improvement between competing refinements rather than the absolute score values. Consequently, small biases in the discriminator are unlikely to accumulate across the iterative reasoning process.

For the discriminator to Dice score correlation analysis, we added the following explanatory text to accompany supplementary Table 6:

Similar behaviour was observed across all evaluated tasks. When using eight adaptation samples, the Pearson correlation between discriminator score and Dice performance was >0.78 for all evaluated tasks, indicating that the learned reward signal remained consistently aligned with the true task objective across domains.

Comment: The refinement action is based on the XOR flipping. This strategy forces the reasoning process to solely operate on hard binary masks, which may lose valuable uncertainty information. Probability-based approaches may be more reasonable for medical image segmentation rather than the proposed the hard-decision logic.

Response: We thank the reviewer for this thoughtful observation. The use of XOR-based refinement was chosen as a deliberate design choice to maintain a tractable action space and a stable optimisation signal during the iterative reasoning process.

Allowing refinements over continuous probability values would substantially increase the complexity of the action space. Instead of deciding whether a pixel should be included or excluded from the segmentation, the agent would need to determine the magnitude of continuous updates across the mask. This would substantially increase the complexity of the optimisation problem, leading to less reliable convergence within practical compute budgets.

Furthermore, continuous values in the segmentation would require the discriminator to interpret subtle differences in probabilities rather than evaluating clearly defined alternatives. The binary XOR-based refinement produces two unambiguous candidate solutions consisting only of binary values at each iteration. For continuous values, the complexity of inputs to the discriminator would increase, resulting in a more difficult task for the discriminator.

Importantly, the use of binary refinements does not eliminate uncertainty information. The

System I module produces probabilistic predictions before thresholding to obtain the initial mask, and System II reasoning generates a trajectory of refinements over successive iterations. By analysing the frequency and spatial distribution of these refinements, it is possible to construct uncertainty maps that highlight regions where the reasoning process repeatedly modified the segmentation, corresponding to high uncertainty.

In this work, we chose and developed the binary refinement formulation as a practical and stable mechanism for iterative reasoning under a learned reward signal. While extending the framework to operate directly on probabilistic mask representations remains outside the scope of this paper, it could be an interesting direction for future work.

Action: To the supplementary section 'The definition of refinement', we added the following:

Our implementation performs refinement at the pixel level using XOR flipping to allow maximum flexibility in the evolving solution space. This binary formulation keeps the refinement action space tractable during iterative reasoning, as the agent only decides whether pixels should be included or excluded from the segmentation rather than determining continuous probability updates. This reduces optimisation complexity and enables stable reinforcement learning convergence, particularly when compute budgets are fixed.

To the same section, we also added the following:

Although refinements operate on binary masks, uncertainty information can still be inferred from the trajectory of refinements across iterations. Regions that undergo frequent modification during the reasoning process correspond to areas of higher ambiguity in the segmentation.

Comment: Will the proposed method handle multi-object segmentation tasks? For instance, when a refinement improves on lesion but degrades another in a multi-lesion image, what will the proposed System II do?

Response: We thank the reviewer for raising this important question. The proposed framework naturally handles multi-object segmentation because the optimisation objective in System II is defined by the discriminator score, which evaluates the overall segmentation quality of the entire mask rather than individual lesions independently.

During both cross-task pre-training and task-specific adaptation, the discriminator is exposed to datasets containing multiple objects within a single image. For example, several datasets used during cross-task training involve multi-structure segmentation tasks (e.g., vessel datasets), and the liver tumour dataset used in our experiments frequently contains multiple lesions per image. As a result, the discriminator learns to evaluate segmentation quality at the level of the full segmentation mask rather than individual objects.

During System II reasoning, candidate refinements are compared using this global discriminator score. If a refinement improves one lesion while degrading another, the discriminator evaluates the net effect on overall segmentation quality. The competing refinement with the higher overall score is therefore selected and enacted. In this way, the refinement process naturally favours modifications that improve the segmentation of multiple objects jointly rather than optimising individual regions in isolation.

Consequently, the framework does not assume a single-object setting and can operate on segmentation masks containing multiple objects, provided such structures are represented in the cross-task training or adaptation data used to calibrate the discriminator.

Action: We have now added the following to the 'The definition of refinement' section in the supplementary materials:

The refinement process operates on the full segmentation mask and does not assume a single-object setting. When multiple objects are present, the distribution-discriminator evaluates the overall segmentation quality across the entire mask. Consequently, candidate refinements that improve some regions while degrading others are compared based on their net effect on the discriminator score, and the refinement yielding the higher overall score is enacted. Because the discriminator is trained on datasets containing multiple objects during cross-task pre-training and task-specific adaptation, it naturally learns to evaluate aggregate or global segmentation quality, even in multi-object scenarios.

Comment: The proposed System II is strongly similar to the Test-time Adaptation approaches. Authors should clarify the theoretical difference between these two frameworks.

Response: We thank the reviewer for this insightful observation. While both approaches operate during inference, the proposed System II reasoning framework differs conceptually from test-time adaptation (TTA) in its optimisation target. TTA adapts the model parameters at inference, while System II optimises over solution hypotheses, as described below.

TTA methods typically update the parameters of the predictive model itself during inference in order to reduce distribution shift between training and test data, often using self-supervised objectives or entropy minimisation. In contrast, in our framework the System I module (task-predictor and distribution-discriminator) remains fixed during System II reasoning. Instead, System II updates a reinforcement learning policy that proposes refinements to the current segmentation hypothesis.

Consequently, the optimisation occurs over the solution space rather than the model parameter space, where the RL agents learn to iteratively refine candidate segmentations guided by the discriminator score. In this sense, System II reasoning performs inference-time optimisation over segmentation hypotheses rather than adapting the segmentation model itself to the test distribution.

We have clarified this distinction in the revised manuscript, as outlined below.

Action: To the section 'Progress towards inference-time compute capabilities in machines', we added the following:

Although System II reasoning operates during inference, it differs fundamentally from test-time adaptation approaches. Test-time adaptation typically updates the parameters of the predictive model to better match the test distribution. In contrast, in our framework the System I networks remain fixed, while the System II module optimises a reinforcement learning policy that iteratively refines candidate segmentations. The optimisation therefore occurs in the solution space rather than through adaptation of the segmentation model parameters.

Comment: From my perspective, the refinement process in System II initializes from the System I's prediction. In extreme cases where the prediction of System I is completely misaligned from the true label, will the XOR refinement operation recover this low-quality first-stage result?

Response: We thank the reviewer for this important question. While System II reasoning is initialised from the System I prediction, the optimisation objective during reasoning is defined solely by the discriminator score. Consequently, the refinement process is not constrained to remain close to the initial prediction and instead searches for segmentations that maximise the discriminator's evaluation of segmentation quality.

The System I prediction primarily serves as an efficient starting point that places the reasoning process near plausible solutions in the segmentation space. In principle, the refinement process could also begin from alternative initialisations (e.g., empty or random masks), as the optimisation objective remains unchanged. However, starting from the System I prediction significantly improves convergence speed because it provides a reasonable initial hypothesis for the reasoning process (as evaluated in preliminary experiments, the per-sample inference time for an empty initialisation was ≈200 hours, compared to ≈40 hours with the System I initialisation).

Therefore, while the quality of the System I prediction can influence the efficiency of convergence, the System II module is designed to iteratively explore alternative segmentations guided by the discriminator score, allowing the refinement process to move away from an initially inaccurate prediction when higher-scoring solutions are identified.

Action: To 'The System II module - for an iterative competition between solution refinements' section in the supplementary materials, we added the following:

System II refinement is initialised from the prediction of the System I task-predictor to provide a reasonable starting hypothesis for the iterative optimisation process. However, the refinement objective is defined solely by the discriminator score, and therefore the reasoning process is not constrained to remain close to the initial prediction. Instead, candidate refinements are iteratively explored and selected based on their impact on the discriminator score.

Comment: Authors do not discuss failure cases, such as cases where System II degrades the initial System I prediction. Such analysis will help us build a more comprehensive understanding of the proposed method.

Response: We thank the reviewer for this helpful suggestion. We agree that discussing potential failure modes can provide additional insight into the behaviour of the framework.

One potential limitation arises from the optimisation relying on the discriminator score as a proxy for the true segmentation objective. Occasional discrepancies between the discriminator score and the true task metric (e.g., Dice/IoU) may lead to refinements that improve the proxy score without improving the true segmentation quality. In practice, this risk is mitigated through task-specific adaptation of the discriminator using labelled samples from the target domain, as well as through the use of relative comparison-based rewards, which reduce sensitivity to biases in absolute discriminator scores. We further evaluated this alignment through correlation analyses between discriminator scores and the true task objective (supplementary Table 6). These analyses show strong correlation for adaptation sets of eight samples or more. However, in tasks that differ substantially from the distribution encountered during cross-task training, such discrepancies may become more pronounced, requiring larger adaptation sets.

A second potential failure mode arises when the initial System I prediction is extremely poor. In such cases, the refinement process may require a large number of iterations before approaching a correct solution, and convergence may therefore be limited by practical inference-time compute budgets. While adaptation helps align the initial System I prediction with the

target domain, this mechanism may be less effective when the appearance of test samples differs very substantially from the limited adaptation data.

Finally, discriminator performance may be influenced by the distribution of structures present in the adaptation samples. For example, if the adaptation set contains predominantly large lesions, the discriminator may initially favour segmentations consistent with that distribution, potentially reducing performance on substantially smaller lesions. Cross-task pre-training across diverse anatomical structures helps mitigate this effect, but some sensitivity to the composition of the adaptation set may remain when only a very small number of labelled samples are available (i.e., <8). In our testing, the correlation between the discriminator score and task metric computed over the test set becomes strong (Pearson's correlation coefficient >0.7) when >8 adaptation samples are available.

Overall, while these scenarios were uncommon in our experiments, they highlight the importance of sufficient adaptation data and careful alignment between the learned discriminator and the true task objective when applying the framework in highly data-constrained or out-of-distribution settings.

Action: We added the following paragraph to 'The abilities of the proposed inference-time compute framework' section in the manuscript:

Although the proposed framework demonstrated strong performance across the evaluated tasks, several potential failure modes are worth noting. First, because System II optimisation uses the discriminator score as a learned proxy for the true task objective, occasional discrepancies between the discriminator and the true metric (e.g., Dice or IoU) could lead to refinements that improve the proxy score without improving the underlying segmentation quality. Second, when the initial System I prediction is extremely poor, a larger number of refinement iterations may be required to approach an accurate solution, potentially exceeding practical inference-time compute budgets in our applications. Third, the discriminator is adapted using a small set of labelled samples from the target domain, and if this adaptation set is unrepresentative (for example containing predominantly large lesions), performance on substantially different structures may be affected. Cross-task pre-training substantially mitigates this risk by making the reasoning process less sensitive to the particular small adaptation set used. While some performance degradation may still arise in the presence of highly atypical outlier data, this is unlikely to affect a whole adaptation set in practice, and can be further minimised through appropriate adaptation-set design and selection.

Comment: Authors have currently provided a working demo for MNIST digit classification (Fig 3 in the revised paper). A README file with sufficient instruction for installing and reproducing this result is provided. I am able to successfully run the provided scripts and I believe the code will be useful for the research community.

Response: We thank the reviewer for verifying the code and for the positive feedback regarding its usability and reproducibility.

Reviewer 2

Comment: I applaud and thank the authors for their in-depth, comprehensive responses to my feedback / that of other reviews – and I am pleased to report that on nearly all fronts, I am amply satisfied with the revisions! The sensitivity sweeps in particular very much deepen my confidence in the robustness of the standout results. While I would still take some issue with the System-1 / System-2 framing, I also recognize this is a popularly-used metaphor in the AI/ML literature and don't think that particular gripe clouds the many other excellent contributions of this work, nor overshadows the better scoping the authors provide in this revision. And so, taken together, I recommend accept as-is! Congratulations to the authors on a job well done.

Response: We thank the reviewer for their thoughtful evaluation of the revised manuscript and for their constructive feedback throughout the review process. We greatly appreciate their positive assessment of the revisions and the additional robustness analyses.

Comment: The authors have adequately addressed the points raised on reproducibility in my primary review. And so, while I was again not able to reproduce the manuscript results from the provided GitHub repo, I accept the authors' declaration of intent to provide more comprehensively reproducible updates on publication.

Response: We thank the reviewer for their careful assessment of the reproducibility aspects of the work. As noted, we are committed to releasing updates upon acceptance/ publication, including full training scripts, data loaders, and hyper-parameter configurations.

Reasoning in machine vision by learning fast and slow thinking
Point-by-point response to reviews

We thank the reviewers and the editor for their constructive feedback in this round of review, and for the recommendation to publish our work.

We address the remaining points from Reviewer 1 in a point-by-point manner below, outlining the 'comment', 'response' and/or 'action'. Editorial requests are addressed in the attached Author Checklist.

Reviewer 1

Comment: Thank you for the clarification, which addressed several of my earlier concerns. A few minor points remain.

Response: We thank the reviewer for their continued engagement with our work. The remaining minor points are addressed below in a point-by-point manner.

Comment: The +1/-1 relative reward scheme may mitigate absolute drift, but it risks creating a severe information bottleneck. By discretizing the reward signal, the framework loses gradient resolution - a policy update that dramatically improves the mask is treated identically to one yielding marginal gains. This could lead to substantial inefficiency in training. It is unclear whether the current framework has any mechanism to recover from this.

Action: To the supplementary materials section 'The System II module - for an iterative competition between solution refinements', we have now added the following:

Although the reward is discretised, the magnitude of improvement is implicitly preserved through selection frequency: refinements that yield larger improvements are more consistently chosen across iterations, allowing their effects to accumulate over time. This common comparison-based RL formulation retains robustness to noise in the discriminator by avoiding reliance on absolute score differences, while still favouring substantially better policies through repeated selection.

Comment: The refinement procedure bears a close resemblance to iterative denoising in conditional diffusion models. The authors should provide theoretical or empirical justification for why the added complexity of a GAN+RL framework offers a meaningful advantage over a simpler test-time conditional diffusion process initialized on System I's output.

Action: To the 'Discussion', we have now added the following:

The proposed refinement process shares some similarities with iterative denoising in diffusion models, however, the underlying mechanism is fundamentally different. Diffusion models learn a fixed reverse process from large datasets that maps noisy inputs to the data distribution,

and apply this process at inference. In contrast, our framework performs per-sample optimisation guided by a learned discriminator, where candidate refinements are iteratively generated, evaluated, and selected. This enables adaptive inference-time refinement conditioned on the specific sample and task objective, allowing direct per-sample optimisation rather than following a predefined denoising path.

Comment: The choice of N=2 agents warrants explicit discussion. The authors should clarify whether this is an architectural constraint or an empirically motivated decision, and address whether scaling to a larger agent pool degrades performance.

Action: To the supplementary materials section 'The System II module - for an iterative competition between solution refinements', we have now added the following:

Limiting the process to pairwise competition, rather than multiple simultaneous comparisons, maintains a well-conditioned optimisation signal (less impacted by absolute discriminator scores) by avoiding the need to rank or weight multiple competing refinements. Multiple comparisons would increase sensitivity to noise in the discriminator scores, requiring ranking or weighting, leading to less stable optimisation. While such extensions are possible in principle, pairwise comparison provides a more reliable optimisation strategy under a learned reward function.

Comment: The robustness of the System I/II adaptation to sample quality remains unverified. Reporting performance standard deviation across multiple sampling seeds would help distinguish genuine improvement from favorable few-shot selection.

Action: We reported standard deviation across different seeded runs in our ablation studies presented in Tab. 5. This was indicated in the caption originally as '*standard deviation is across different runs*', which we have now updated to the following, for further clarity:

standard deviation is reported to measure variance across 4 runs with different seeds

Comment: I would like to kindly note that the public repository has not been updated since the last round of review. I would strongly encourage the authors to release the full codebase upon acceptance, enabling the community to reproduce all reported experiments — beyond the existing MNIST demo.

Action: We have now added a sub-directory in the repository titled 'experiment_example', which contains example data, network definitions, training script and data loader to allow the medical image segmentation described in the paper. The datasets are available from the sources outlined in Tab. 1 and hyper-parameters for final runs are available in Tab. 2.
